# Optimized CUT&RUN protocol for activated primary mouse B cells

**DOI:** 10.1371/journal.pone.0322139

**Published:** 2025-04-24

**Authors:** Stormy E. Ruiz, Robert W. Maul, Patricia J. Gearhart

**Affiliations:** 1 Laboratory of Molecular Biology and Immunology, National Institute on Aging, National Institutes of Health, Baltimore, Maryland, United States of America; 2 Graduate Program in Immunology, Johns Hopkins University School of Medicine, Baltimore, Maryland, United States of America; Sichuan University, CHINA

## Abstract

ChIP-seq has long been the standard for study of chromatin-protein interactions. However, development of a new technique, CUT&RUN, showed substantial advantages compared to ChIP-seq including higher quality signal while using substantially less sample. While a powerful technique, the original protocol was designed using cell lines and histones as targets. Due to their fragility, this was unsuitable for obtaining high-quality data from activated primary B lymphocytes. To adapt this protocol for B cells, cells were fixed prior to nuclear isolation, and several critical adjustments were introduced to the procedure and reagents. We measured binding of H3K4me3 histone and RNA Polymerase II, detecting robust peaks with as little as 100k nuclei. Additionally, freeze-thaw of B cells prior to processing did not affect results, emphasizing the flexibility of this modified technique. Using the protocol described here will allow one to quantify non-histone proteins bound to DNA from limited numbers of B cells with more efficiency than can be achieved from the current standard, ChIP-seq.

## Introduction

The study of chromatin-interacting proteins and their modifications is currently a substantially growing field for studying biological variation, and the techniques used to investigate them have rapidly evolved and advanced over the past several years. The standard technique for measuring protein localization to DNA is Chromatin immunoprecipitation (ChIP). When coupled with next-generation sequencing (ChIP-seq), high levels of protein localization can be identified across the entire genome. However, this technique has limitations, specifically the ability to perform reproducible ChIP-seq experiments when cell numbers are limited. Thus, the development of Cleavage Under Targets and Release Using Nuclease (CUT&RUN) [1,2] opened a new opportunity for studying chromatin-interacting proteins from limited cell samples. A technique similar to ChIP-seq, CUT&RUN analyzes chromatin-interacting proteins by sequencing of DNA fragments. The main differences are that ChIP-seq uses sonication or enzymatic digestion to generate fragments surrounding protein-DNA complexes and subsequent capture of protein by immunoprecipitation. These methods can be inconsistent and biased and can destroy large protein complexes [3–8]. CUT&RUN detects a specific footprint of where a protein of interest is bound by generating fragments using an antibody-bound nuclease and collection of the fragments by heat and diffusion. The specificity of cutting chromatin at targets of interest greatly reduces the amount of genomic material that is needed for sequencing, particularly for untreated inputs needed to determine a pulldown “enrichment” for peak calling. Instead, CUT&RUN uses IgG negative control samples to determine off-target cleavage of chromatin but do not require deep sequencing as ChIP-seq inputs do. CUT&RUN has a low background and high signal-to-noise ratio due to this targeted cutting. This enables CUT&RUN experiments and its sister technique, Cleavage Under Targets and Tagmentation (CUT&TAG), to sample much smaller populations of cells, with some even achieving success with multiple targets in individual cells [9–11].

The original CUT&RUN technique works by taking cells or nuclei and capturing them with Concanavalin A magnetic beads. The sample cells/nuclei are then incubated with antibody against the target of interest, washed to remove excess antibody, and incubated with Micrococcal Nuclease (MNase) tagged with Protein A/G (pAG). This tag binds to the target antibody and non-bound MNase is washed away. The sample is then spiked with calcium chloride which activates the MNase, digesting the nearby DNA and freeing the fragment of DNA protected by the protein(s) of interest. These fragments diffuse out of the cell/nuclei upon heating and can then be collected from the supernatant and further processed for library preparation and sequencing.

When looking to study target proteins in ex vivo-stimulated primary B lymphocytes, the original CUT&RUN protocol was unsuitable, and there is no report to date of a complete and consistent protocol for non-histone proteins for primary B cells publicly available. Some of the challenges with these cells include: 1) the potential activation of lymphocytes by Concanavalin A (ConA) beads [12–14], 2) activation of surface-bound B cell receptors, and 3) the inherent cellular fragility of activated B cells [15–19]. During the CUT&RUN procedure, proteins are detected using target antibodies which direct the nuclease to the target protein on the chromatin. Since B cells produce endogenous antibodies (in the form of membrane-bound B cell receptors or soluble secreted antibody), there is potential for the endogenous antibody to interfere with the nuclease recruitment to the target antibody, reducing the efficiency of DNA fragmentation. Activated B cells and their isolated nuclei are fragile and primed for death without adequate survival signals [15–19] which makes native CUT&RUN a challenge, particularly for samples which require extensive preparation by time and handling, such as isolation of primary B cells from animals and subsequent fluorescence-activated cell sorting of very specific populations of interest. Due to this stability issue, non-histone targets are much more difficult to reliably probe, and thus signals and consistency across experiments is very low.

To circumvent these issues, the CUT&RUN protocol was modified at several key points. To overcome the cellular activation and endogenous antibody interference, B cell nuclei were used instead of whole cells, eliminating the cells’ ability to signal and clear surface antibody. This step also gives the extra benefit of removing cytosolic proteins, which may affect strength of signals for genome bound targets that are present at higher concentrations in the cytosol than the nucleus. A gentle fixation was added, and reagents and steps were adjusted to stabilize the nuclei and chromatin-protein interactions [20]. With this modified protocol, robust and replicable data was obtained from activated B cells using a varying number of input nuclei. This technique also allows for the use of either freshly isolated or frozen nuclei, with little to no impact on result consistency, making this protocol more convenient and flexible.

## Materials and methods

### Resource availability

#### Lead contact.

Further information and requests for resources and reagents should be directed to and will be fulfilled by the lead contact, Patricia J. Gearhart (gearhartp@nih.gov).

#### Materials availability.

C57BL/6J-*Igh*^em1Pjg^ mice are available upon request.

A complete table of all resources used in this study can be found in Supporting Information File 2.

### Experimental model

#### Mice.

Male and female C57BL/6JN or 2-2J1 mice (C57BL/6J-Igh^em1Pjg^) 8–20 weeks of age were used in this study. Mice are housed under standard care conditions and managed by the Comparative Medicine Section at the National Institute on Aging Baltimore Animal Facility. Animal protocols were reviewed and approved by the Animal Care and Use Committee of the National Institute on Aging (ASP 281-LMBI-2025).

### Method details

#### Protocol.

The protocol described in this peer-reviewed article is published on protocols.io at dx.doi.org/10.17504/protocols.io.3byl491y2go5/v1 and is included for printing as Supporting Information File 1 with this article.

#### B cell culture stimulation.

Spleens from naïve mice were isolated and crushed through a 70 µm filter to form a single cell suspension. Red blood cell lysis was performed using ACK lysis buffer and follicular B cells were purified by positive selection using CD23 magnetic beads (Miltenyi Biotec) over an LS column (Miltenyi Biotec). B cells were cultured in RPMI 1640 (Gibco) supplemented with 10% FBS (Gibco), 1% GlutaMAX (Gibco), 1% Pen/Strep (Gibco), and 50 µM betamercaptoethanol and stimulated using 10 µg/mL lipopolysaccharide (Millipore Sigma) and 10 ng/mL interleukin-4 (R&D Systems). Cells were plated at a density of 3.125 x 10^5^ cells/mL using a T-75 flat culture flask and incubated at 37°C with 5% CO_2_ for 48 hours without disturbance. When plating, one milliliter of cells + media + cytokine was separately added to a 24-well culture plate and incubated for 72 hours to validate success of stimulation by percent of IgG1^+^ live cells (>20%).

#### Freeze-thaw of cultured primary B cells.

Cells cultured for 48 hours were collected, centrifuged at 600xg for 5 minutes, and resuspended in 100% FBS at room temperature (RT). DMSO was added to a final concentration of 10%, sample was briefly mixed, and then immediately placed into a Mr. Frosty™ freezing container and stored at -80°C which allows cooling and freezing of samples at a rate of approximately 1°C per minute. Sample tubes were transferred to standard boxes after complete freezing. To thaw, the tube was removed from the -80°C freezer and dipped into a 37°C water bath with gentle agitation to quickly thaw. Cells were then immediately centrifuged at 600xg for 5 minutes and resuspended in RT Dulbecco’s phosphate buffered solution (DPBS) or PBS +10% FBS +2mM EDTA (PBS-FE) for counting and then processed for dead cell removal.

#### Dead cell removal.

48-hour stimulated cells were removed from culture by pipetting and were washed once with RT DPBS or PBS-FE. Cells were centrifuged at 600xg for 5 minutes and resuspended in dead cell removal beads (Miltenyi Biotec) at a volume according to manufacturer’s protocol. Sample was incubated at RT for 15 minutes, and then volume was adjusted to 500 µL using DPBS or PBS-FE. Sample was applied to a magnetic LS column (Miltenyi Biotec) and live cells were collected from the flow through. The column was washed twice using 7 mL of DPBS or PBS-FE. Live cells were counted using trypan blue and a standard light microscope.

#### Nuclei preparation.

Live cells were fixed in DPBS or PBS-FE directly after purification at RT by addition of formaldehyde to a 0.1% or 1% final concentration. Cells were mixed by gentle and continuous inversion for 1 or 10 minutes, and then the reaction was immediately quenched with the addition of glycine to a final concentration of 0.125 M. Cells were washed 3 times using cold DPBS or PBS-FE and the sample was maintained on ice for the remainder of the experiment. After the final wash, the cell pellet was gently resuspended in nuclei isolation buffer at 1–5 x 10^6^ cells/mL (see S3 Text for recipes) and incubated on ice for 10 minutes. Nuclei were then centrifuged at 600xg for 5 minutes and resuspended in 1–2 mL Wash XLS buffer (see S3 Text for recipes). A small aliquot (5–20 µL) of nuclei were analyzed for quality and counted with trypan blue staining under a standard light microscope. Nuclei were once more centrifuged at 600xg for 5 minutes and resuspended to a final concentration of C x10/mL where C = the number of nuclei intended for use for each CUT&RUN sample (ex. 1 x 10^6^ nuclei/mL for samples of 1 x 10^5^). If the nuclei sample contained excessive trypan blue-stained debris, additional washes using Wash XLS buffer were performed and reassessed on the microscope. Volume of nuclei needed for all samples was aliquoted into a separate microcentrifuge tube on ice.

#### Binding of nuclei to Concanavalin A magnetic beads.

Concanavalin A magnetic beads were thoroughly mixed and 10 µL of beads per sample were transferred into a PCR-sized tube (EpiCypher). Tube was placed on a magnetic stand (10x Genomics) and supernatant was removed. Beads were washed using Activation buffer at least 2 times with thorough mixing between each wash. Beads were then resuspended in Activation buffer at 10 µL per 10 µL of beads used. Prepared beads were added directly to aliquoted nuclei, mixed by inversion and briefly centrifuged to collect liquid, and incubated on ice for 30 minutes. 110 µL of nuclei + bead mixture was aliquoted into 8-strip PCR tubes on ice. Quality of nuclei was assessed by trypan blue staining (see step-by-step protocol in S1 Protocol). Strip tubes were placed on magnetic rack and allowed to clear. Working one tube at a time, supernatant was discarded and 50 µL of Antibody Binding Buffer was added without mixing. Strips were then mixed by turning tubes horizontally and gently shaking back and forth along the long side of the tubes and then briefly centrifuged to collect liquid (to prevent sample loss by pipetting) and placed on ice.

#### CUT&RUN.

Protocol uses EpiCypher ChIC/CUT&RUN kit and materials, and steps were followed unless described here. A detailed step-by-step protocol can be found in supplemental materials. Antibody Binding buffer uses 1 mM spermidine (see S3 Text for recipes). 1 µg of antibody against the target of interest was used for each sample. Strip tubes were angled 45° with caps tilted upwards (to prevent sample collecting in cap) and shaken at 800 rpm at 4°C overnight (16 hours) (S5 Fig). Strips were placed on magnetic rack to clear. Working tube by tube, supernatant was removed and beads were washed twice using 200 µL Wash XLS buffer on magnetic rack and 50 µL of Wash XLS was added. 2.5 µL of pAG-MNase was added, tubes were mixed by gentle shaking and quick spin, and incubated on ice for 30 minutes. Strips were placed on magnetic rack to clear. Working tube by tube, supernatant was removed and beads were washed twice using 200 µL Wash XLS buffer on magnetic rack and 50 µL of Wash XLS was added. Tubes were mixed by gentle shaking and quick spin and placed on ice. Then, 1 µL of 100 mM Calcium Chloride was added to each sample. Samples were wrapped in plastic wrap or placed in a bag and buried in wet ice and incubated for 2 hours. Stop buffer + normalization spike-in according to number of nuclei used was mixed and 33 µL was added to each sample. Strips were then removed from ice and placed in a pre-heated thermocycler (Applied Biosystems) set to 37°C for 10 minutes. Tubes were placed on magnetic rack and cleared, and supernatant was transferred to new strip tubes. 1.6 µL of 5% SDS and 1 µL of Proteinase K (Ambion) were added, and samples were incubated at 55°C overnight (16 hours) in a thermocycler. Fragments were purified using spin column reagents supplied from ChIC/CUT&RUN kit (V3 kit or older) or SPRI Select beads (V4 kit or newer, or Beckman Coulter) at a 1.4x concentration (120 µL of beads) and eluted in 25 µL of 0.1x TE buffer. Samples were stored at -20°C until library preparation.

#### Library preparation.

Library Preparation was performed using EpiCypher Library Prep kit according to manufacturer instructions unless described here. Samples were not initially quantified by Qubit and diluted and instead all 25 µL from the previous step was used for end repair. Adapter was diluted 0.5x using 0.1x TE buffer and 1.25 µL of this diluted adapter was used for the ligation reaction. i7 and i5 indexing primers were diluted to 0.2x using molecular biology-grade water before use and 1 µL of diluted primers was used for each reaction. PCR amplification was performed for 16 cycles. Samples were then diluted to 50 µL using 0.1x TE buffer and double-sided SPRI Select purification was performed using 0.65-0.95x concentrations (32.5 µL, then 15 µL beads). Samples were eluted in 30 µL 0.1x TE buffer and stored at -20°C.

#### Library quantification and quality control.

Libraries were quantified using Qubit 1x dsDNA High Sensitivity kit (Invitrogen) with 198 µL of 1x reagent and 2 µL library. Library size was then determined using Agilent Bioanalyzer High Sensitivity DNA kit (Agilent Technologies) according to manufacturer protocol. Libraries were evaluated for single-nucleosome-sized peaks and if needed, further SPRI Select purification was performed and libraries were reassessed until size distribution was about 300–500 base pairs.

#### Sequencing.

Libraries were pooled into equimolar ratios and sequenced by an Illumina MiniSeq or Illumina NovaSeq 6000 using paired-end 300x2 or 200x2 sequencing kits (Illumina), respectively.

#### Data analysis.

Demultiplexing of raw sequencing data was performed using bcl2convert (Illumina). Reads were trimmed using Trim Galore! [21] and aligned to the mm39 mouse genome (NCBI) using bowtie2 [22] in paired-end mode. Sam files were then collated, mates were fixed, sorted, and PCR duplicates were removed using SAMtools [23,24]. MACS2 [25] was used for peak calling using the setting “--broad” for all samples. Peak calling was performed using the bam files twice: once with the corresponding IgG sample bam file as a control (H3K4me3/Pol II with IgG), and once with no control (all samples individually). Peak files generated were used for interpretation for visualization by Integrative Genomics Viewer, (IGV) [26] and quantitative analysis by deeptools and DiffBind. Tracks were visualized using IGV without normalization. Irreproducibility Discovery Rate [27] analysis was performed using IDR with the settings “--use-best-multisummit-IDR” and “--use-nonoverlapping-peaks”. Using deeptools [28], bam files were converted to bigwig format, then genomic heatmaps and profiles were generated for both individual samples and samples plus IgG as a control (by use of “bamCompare” versus “bamCoverage”). DiffBind [29,30] was used for comparison of groups of datasets (by technique, treatment, input amount) and to generate binding affinity matrices, principal component analysis (PCA) plots, and differential binding profiles. The mm39 blacklist from the Dozmorov lab [31] was applied in DiffBind. Datasets were split into three groups for comparisons: Dataset group 1 includes ChIP-seq, original CUT&RUN, and optimized CUT&RUN using freshly isolated cells from culture, Dataset group 2 which includes experiments with variable fixation of freshly isolated cells from culture, and Dataset group 3 which includes only optimized CUT&RUN datasets (using fresh and freeze-thawed cells). A detailed breakdown of datasets can be found in S4 Table. For dataset group 1: normalization was performed using library sizes. For dataset groups 2 and 3: E. coli spike-in DNA was used for normalization. For all dataset groups: The analysis in DiffBind was performed twice: the first is where the sample sheet included corresponding IgG samples in the “bamControl” and “ControlID” columns and the column for peak files used the bedgraph/xls files generated by MACS2 with IgG a control. The second used a sample sheet excluding the “bamControl” and “ControlID” columns and instead IgG experiments were identified as individual samples, and the bedgraph/xls peak file was generated in MACS without the use of an optional control.

#### Figure generation.

Figures were generated using IGV, IDR, deeptools, R and R studio, DiffBind, and BioRender.

#### Quantification and statistical analysis.

IDR analysis was performed to compare samples from either the same set of experiments using different number of nuclei or from independent experiments using the same number of nuclei. The cutoff for statistical significance in the IDR analysis was a log10 score < 0.05. Fraction of reads in peaks (FRiP) percentages were calculated in DiffBind using control samples and normalization applied. Uniquely aligned reads were counted using SAMtools and identified peaks were counted from MACS2. All calculated values can be found in S4 Table.

## Results

### B cell-adapted CUT&RUN protocol shows improvement over original technique

Using the original CUT&RUN protocol on activated B cells showed poor reproducibility as over half of the experiments resulted in the breakdown of all nuclei upon introduction to ConA beads. For the few experiments in which the nuclei stayed intact for the entire procedure, downstream data analysis showed only peaks for the histone mark H3K4me3 could be reliably detected, while those for RNA Polymerase (Pol) II were poor. Thus, a modified version of CUT&RUN needed to be developed for primary B lymphocytes where the integrity of the sample was maintained, and where we could reliably capture non-histone targets.

**Fig 1:**
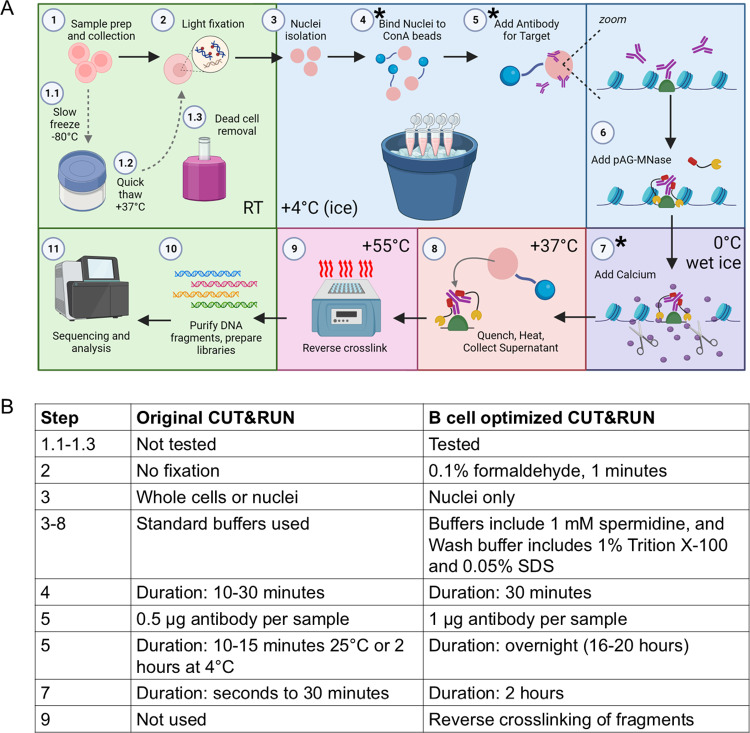
Summary of modified CUT&RUN protocol for B cells. A: Prepared cells can either be used fresh (step 1 to step 2) or frozen (step 1 to steps 1.1, 1.2, 1.3, 2). Briefly, cells are lightly fixed and intact nuclei are isolated. Nuclei are bound to ConA beads and then incubated with antibody overnight. pAG-MNase is bound and then activated with calcium to initiate digestion. Fragments are collected via heating of sample and further purified after reverse-crosslinking. Fragments are then used to prepare libraries and for subsequent sequencing. Steps indicated with an asterisk (*) need extended incubation times. Created in BioRender. Maul, R. (2023) BioRender.com/z84l302. B: Table summarizing key differences in this protocol compared to traditional CUT&RUN protocols.

**Fig 2:**
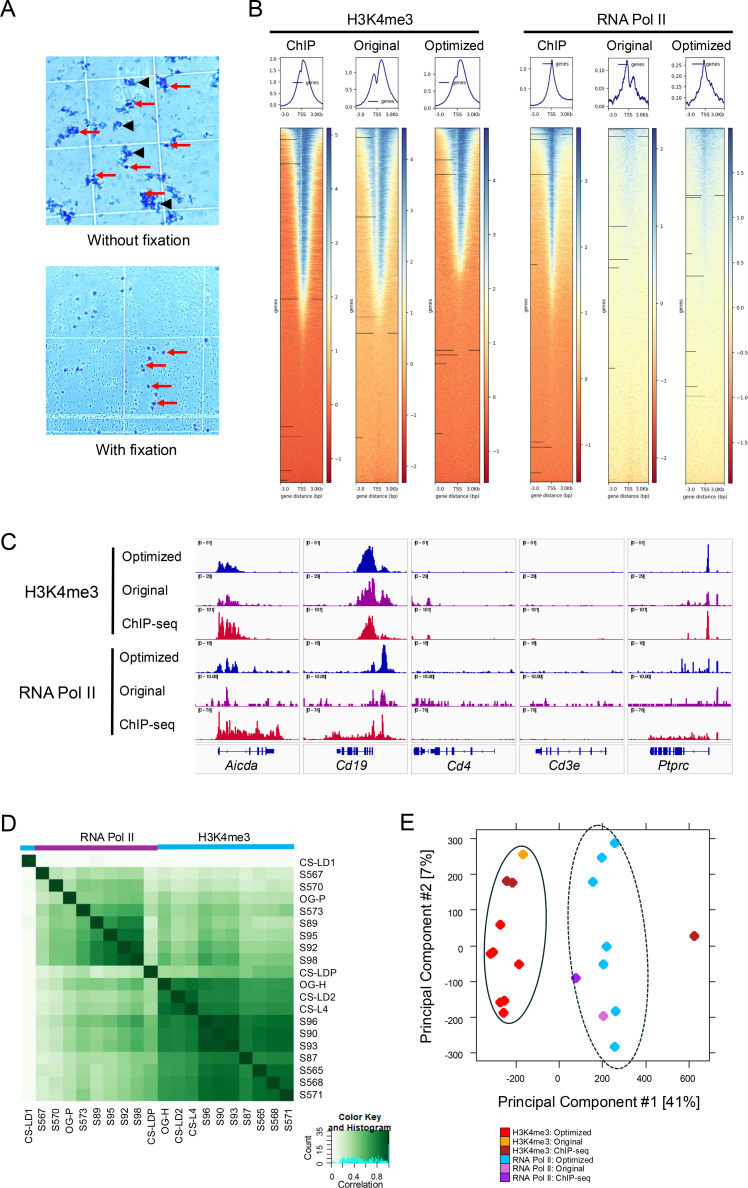
Optimized CUT&RUN method performs better than the original CUT&RUN and ChIP-seq. A: An equal number of nuclei (500k) were bound to 10 µL of prepared ConA beads. Nuclei were either not fixed (top) or nuclei were fixed and used buffer including higher spermidine concentration (bottom). Nuclei in the no fixation condition tend to fall apart into disperse debris which precipitates further nuclear loss. Nuclei that were fixed and with higher spermidine concentrations remain intact once introduced to ConA beads. Select intact nuclei are indicated by red arrows while black arrowheads indicate disrupted nuclei and nuclear debris. B: Genomic heatmaps and profiles showing aligned sequences from ChIP-seq (ChIP), standard CUT&RUN (Original), and B cell-modified CUT&RUN (Optimized) for H3K4me3 and RNA Pol II. Shown is signals +/- 3 kb upstream and downstream from TSSs C: Tracks for H3K4me3 and RNA Pol II at select genes from ChIP-seq, standard CUT&RUN (Original), and B cell-modified CUT&RUN (Optimized). Traces shown are not normalized but instead auto-scaled to display features. D: Affinity matrix comparing all normalized replicates from ChIP-seq, standard CUT&RUN, and B cell-modified CUT&RUN experiments for H3K4me3 (n=3, 1, 7, respectively) and RNA Pol II (n=1, 1, 7, respectively). Datasets for ChIP-seq, original, and B cell-optimized CUT&RUN are indicated by the prefixes CS-, OG-, and S-, respectively, and groups by target (H3K4me3 or RNA Pol II) are indicated by the colored bars above. Blue is H3K4me3 and purple is RNA Pol II. E: PCA analysis of peaks from all normalized datasets. The solid oval indicates the H3K4me3 datasets while the dashed oval indicates the RNA Pol II datasets.

After testing several modifications of this technique, an optimized protocol was created and the steps are outlined in Fig 1A with differences highlighted in Fig 1B. *Ex vivo*-cultured primary B cells were collected, and dead cells removed using a magnetic dead cell removal kit. To maintain integrity of the cells, samples were lightly fixed with 0.1% formaldehyde for 1 min in DPBS followed by quenching with glycine and several washes with cold DPBS. Samples were kept on ice for the remainder of the experiment to maintain fixation. Nuclei were isolated using a nuclear isolation buffer with higher concentrations of spermidine to help further stabilize the sample. It was observed that a combination of fixing cells, isolating the nuclei, and using higher spermidine concentrations was more effective and resulted in lower sample loss (Fig 2A and S5). Nuclei were resuspended in a modified wash buffer containing excess spermidine, 0.05% SDS, and 1% Triton X-100, and bound to activated ConA beads. Target antibody was added, and samples were incubated with shaking at a 45-degree angle overnight (see S6 Fig). pAG-MNase was added to each sample and incubated for 30 minutes on ice, which was then followed by washing and the addition of calcium chloride to activate endonuclease activity. Samples were incubated on wet ice for an extended time of 2 hours. The wet ice limits pAG-MNase diffusion, and the extended digestion time ensures complete fragmentation with fewer multinucleosome-sized fragments. Samples were heated and fragments collected, at which point they were reverse-crosslinked overnight to release chromatin. Fragments were purified, and downstream library preparation was performed.

Datasets obtained using these techniques were used to generate the genomic heatmaps and profiles +/- 3 kb from transcription start sites (TSSs) (Fig 2B, S7 Fig A-C). While H3K4me3 appears similar across both protocols, RNA Pol II was unable to be captured efficiently using the original CUT&RUN. In contrast, with using the modified protocol, there is a characteristic sharp peak located directly over TSSs and a wider density across highly expressed genes (Fig 2B-C, S7 Fig B and D). This was true for whether or not processing used IgG as a negative control in generation of the heatmaps/profiles by deeptools.

To confirm the benefits of the optimized protocol for B cells, H3K4me3 and RNA Pol II localization was compared against results obtained using the original protocol. Examining key positive and negative control genes, both H3K4me3 and RNA Pol II were observed at B cell-expressed genes (*Aicda*, *Cd19*, *Ptprc*), and there was no signal at non-B cell genes (*Cd4*, *Cd3e*) (Fig 2C, S7 Fig D). Importantly, fixation in the adapted CUT&RUN did not appear to alter the distribution of peak data (Fig 2C-E). Of note, RNA Pol II peaks from the original protocol had weak peak signals barely above background and IgG samples, while results from the optimized protocol resulted in the expected signals over TSSs and more easily identifiable peaks at individual genes (Fig 2C, S7 Fig D). By binding affinity matrix of all calculated binding sites, both protocols appear to have good cross-correlation for H3K4me3 and RNA Pol II (Fig 2D, S7 Fig E-F). Principal component analysis (PCA) (Fig 2E, S7 Fig G-H) shows that both H3K4me3 datasets and RNA Pol II datasets produced using CUT&RUN tend to correlate and cluster together well with their respective factor (H3K4me3 or RNA Pol II) groups.

### Confirmation of optimized CUT&RUN data compared to ChIP-seq

To verify the accuracy of results from the optimized protocol, the results for H3K4me3 were compared to previously published ChIP-seq datasets in which H3K4me3 and RNA Pol II localization was mapped in total murine splenic B cells stimulated in culture with LPS + IL-4 or LPS + anti-IgD-dextran for 48 hours [32]. Genomic heatmaps and profiles show that ChIP-seq and B cell-optimized CUT&RUN signals appear similarly around TSSs (Fig 2B, S7 Fig A-C). Comparison of peaks at key B cell genes showed that the optimized CUT&RUN protocol has very similar peak traces to ChIP-seq (Fig 2C, S7 Fig D). All binding sites were compared using a binding affinity matrix which shows good to moderate cross-correlation of peak distribution across techniques (Fig 2D, S7 Fig E-F). While the datasets across techniques do not have very high cross-correlation for RNA Pol II (about 0.4 for ChIP-seq to optimized CUT&RUN), it must be noted that the ChIP-seq experiment was performed under slightly different stimulation conditions (IL-4 + LPS, which gives a stronger activation than IL-4 + αCD40), using total splenic B cell samples, and using a different technique. PCA shows distinct clustering of datasets by factor, particularly by principal component 1 (Fig 2E, S7 Fig G-H). To further examine the extent of genome wide peak similarity, Irreproducibility Discovery Rate (IDR) analysis was performed. This analysis compares the ranking of peaks between the two samples and produces a score representing the likelihood of the peaks being a false discovery [27]. The optimized protocol datasets showed more matched peaks with an IDR <0.05 compared to the ChIP-seq dataset than the original CUT&RUN, showing that this modified CUT&RUN can reproduce ChIP-seq results better than standard CUT&RUN (S8 Fig). Importantly, B cell-optimized CUT&RUN provided comparable results to ChIP-seq despite the 200-fold difference in input sample, highlighting the advantages of the CUT&RUN technique for limited sample numbers.

### Over-fixation results in lower quality datasets

Since fixation is used in this modified B cell CUT&RUN protocol, experiments were performed to compare different levels of fixation. The fixation conditions used were as follows: light (0.1% formaldehyde for 1 minute), medium-low (0.1% formaldehyde for 10 minutes), medium-high (1% formaldehyde for 1 minute), and heavy (1% formaldehyde for 10 minutes). Fixation was performed on cells post-dead cell removal and pre-nuclear isolation. Tubes were continuously and evenly mixed during fixation, and all reactions were quenched with glycine to a final concentration of 125 mM. After fixation, all resulting nuclei and subsequent DNA fragments were processed the same according to the optimized protocol.

Prior to sequencing, the effect of fixation on fragmentation could already be clearly observed by the yield and size distribution (S9 Fig). Despite being processed using the same starting number of input nuclei and same steps, libraries prepared from samples with heavier fixation (by time and/or percentage of fixative) showed a reduction in the total amount of DNA in the size range for efficient sequencing (200–500 base pairs). Extended fixation times resulted in a larger proportion of multinucleosome-sized fragments while higher fixative concentration resulted in a striking loss of recovered fragments. In the case of heavy fixation with extended time and higher fixative concentration, several distinct larger peaks indicating multinucleosome-sized fragments were present that did not appear in the lighter fixation conditions. With heavier fixation conditions, it is probable that cleaved DNA fragments are unable to diffuse out of the nucleus and/or cleavage of the nearest DNA by MNase may be blocked by fixation, leading to larger fragment sizes corresponding to one or more additional nucleosomes. The result is lower yields of high-quality libraries which will certainly affect results, particularly at even lower nuclei input amounts. This is also the trend we observed when digestion is limited to 30 minutes instead of the full 2 hours (under light fixation), which highlights that the timing and degree of fixation is a critical variable that must be considered.

**Fig 3:**
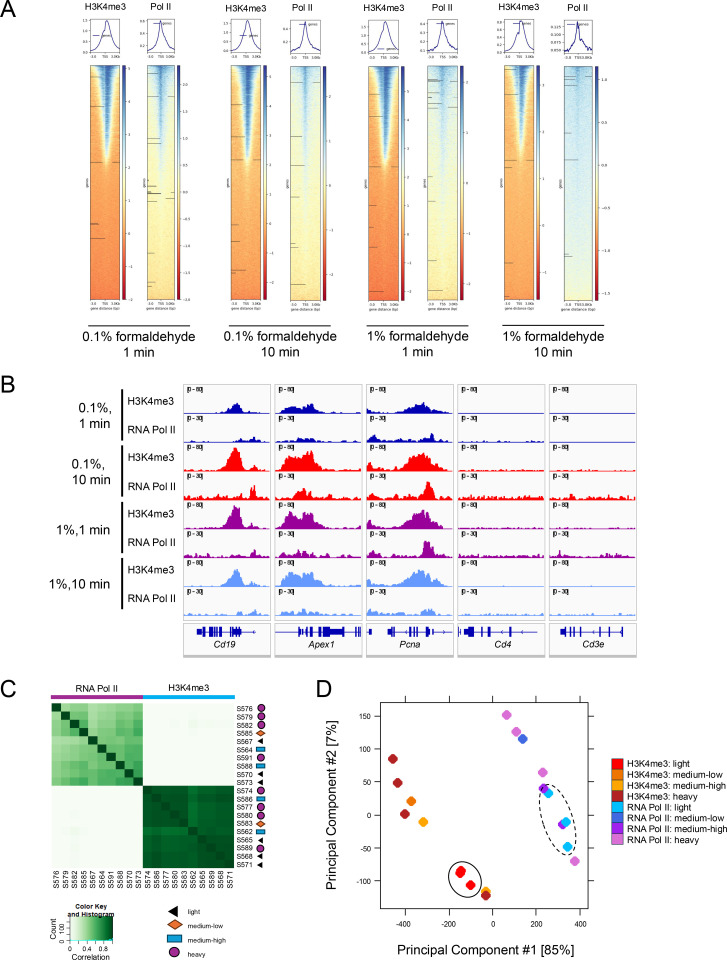
Extended fixation time or increased formaldehyde concentration result in less consistent datasets. A: Genomic heatmaps and profiles for light (0.1% formaldehyde for 1 min), medium-low (0.1% formaldehyde for 10 min), medium-high (1% formaldehyde for 1 min), and heavy (1% formaldehyde for 10 min) fixation conditions prior to nuclei preparation for H3K4me3 and RNA Pol II. B: Tracks for H3K4me3 and RNA Pol II at select B cell (Cd19), treatment-stimulated genes (Apex1, Pcna), or non-B cell genes (Cd4, Cd4e) for the four fixation conditions. C-D: Affinity matrix (C) and PCA plot (D) comparing all normalized sites for H3K4me3 and RNA Pol II for the four fixation conditions. For D, a solid oval indicates the cluster of lightly fixed H3K4me3 datasets while the dashed oval indicates the cluster of lightly fixed RNA Pol II datasets.

Generation of genomic profiles gives an initial view on how varying fixation affects the global distribution of signals. The expected nucleosome displacement is apparent for H3K4me3 with light fixation, however, as the time of fixation is increased, that profile tends to become blunted (Fig 3A, S10 Fig A). For RNA Pol II, it was observed that at longer fixation times, signals are broader and present genome-wide which indicates overfixation. Zooming in on traces for specific positive (*Cd19*, *Apex1*, *Pcna*) and negative (*Cd4*, *Cd3e*) genes, it is clear that stronger fixation conditions result in higher background signals for both RNA Pol II and IgG (Fig 3B, S10 Fig B).

Binding affinity matrices using either all peaks or significantly differential peaks by factor show that signals appear in similar locations across all fixation levels (Fig 3C, S10 Fig C-D). However, PCA shows that fixation conditions tend to group together for each factor. The light fixation-treated samples cluster tightly together for both H3K4me3 and RNA Pol II by principal components 1 and 2 (accounting for 85% and 7% of the variability, respectively), while the stronger fixation-treated samples displayed more variability. This was true for both cases when samples were either analyzed with or without using IgG samples as a control (Fig 3D, S10 Fig E-F). Together, these results suggest there is more consistency for the light fixation group, in addition to higher recovery of fragments mentioned earlier.

### Examining the flexibility of the optimized CUT&RUN protocol

**Fig 4:**
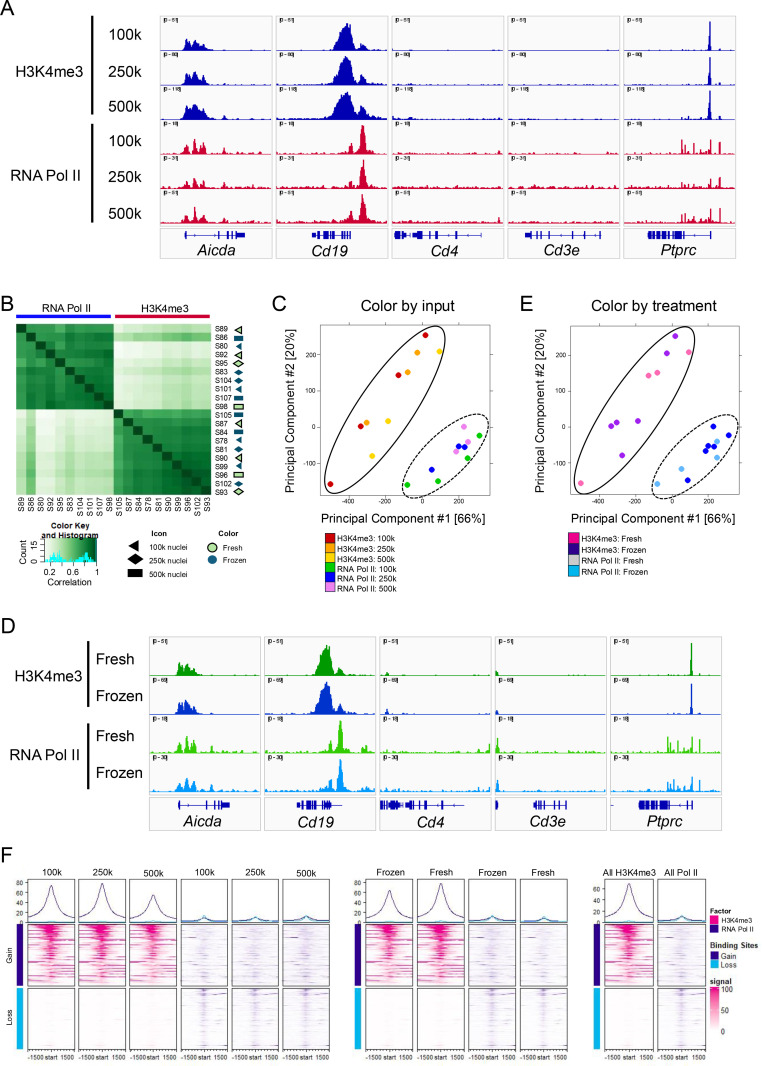
Optimized CUT&RUN method results are consistent at multiple input concentrations. A: Traces where CUT&RUN was performed at input nuclei counts of 100k (n=4), 250k (n=3), or 500k (n=3). Targets probed include H3K4me3 (shown in red) and RNA Pol II (shown in blue). Traces shown are not normalized but instead auto-scaled. B Affinity matrix showing correlation of all normalized samples. Experiments where 100k, 250k, or 500k are indicated by triangle, diamond, or rectangle icons, respectively. Icons colored green indicate cells were used fresh from culture and blue indicated cells were frozen and thawed prior to experiment. C and E: PCA comparison for normalized H3K4me3 and RNA Pol II replicates colored by number of input nuclei (C) or if cells were used fresh or frozen and thawed (E). Solid ovals indicate H3K4me3 datasets and dashed ovals indicate the RNA Pol II datasets. D: Tracks for fresh (n=4) and frozen (n=6) samples at select genes for HK4me3 (green) and RNA Pol II (blue). F: Differential binding profiles and heatmaps for all samples grouped on number of nuclei input (left), fresh or frozen treatment (middle), and factor (right) and displayed +/- 1.5 kb from TSSs.

A major advantage of the CUT&RUN technique is the ability to use lower cell numbers to obtain high quality data. To determine the effect of input number on the effectiveness of the optimized CUT&RUN method, varying inputs of nuclei, 100k, 250k, or 500k, were tested for H3K4me3 and RNA Pol II. A comparison of results for select genes is shown in Fig 4A and S11 Fig A-C. Across these conditions, the peaks appear similarly distributed. The entire datasets were compared using a binding affinity matrix (Fig 4B) and PCA (Fig 4C) which shows very strong correlation of samples within target groups (between 0.6 and 1.0) and importantly – no separate clustering of datasets by the number of nuclei used. Thus, the modifications made in the optimized protocol are sufficient for very low cellular inputs with the results unchanged.

To test the protocol’s experimental flexibility, CUT&RUN was performed on cells which had undergone freeze-thawing. B cells were stimulated in culture for 48 hours and then collected in 100% FBS at 10–20 million cells/mL. DMSO was added to a final concentration of 10% v/v and samples were slow frozen to -80°C (Fig 1 step 1.1) using an isopropanol freezing container. After several days, the sample was quick thawed at 37°C and immediately processed for dead cell removal, light fixation, and then processed using the optimized CUT&RUN protocol (Fig 1 steps 1.2, 1.3, and 2). This freeze-thaw method showed high recovery of live cells, typically 50–80%. We also tested a protocol for freezing prepared nuclei using a buffer containing 10 mM HEPES pH 7, 2 mM MgCl_2_, 25 mM KCl, 250 mM sucrose, 1 mM DTT and 70% glycerol by volume supplemented with protease inhibitor cocktail and freezing directly at -80°C, however we recovered no intact nuclei over several attempts. Fig 4B shows the results for a set of four experiments comparing all datasets where B cell nuclei were prepared from either fresh or frozen and thawed cells. As before with nuclei input amounts, there are not higher correlations from samples that were compared to like treatments (Fresh vs. Fresh, Frozen vs. Frozen). Instead, all datasets within the same target group show very high correlation, regardless of treatment. From the set of genes shown in Fig 4D and S11 Fig A-C, we again observe a high similarity of signal across the two treatment conditions. By PCA where treatment is emphasized by color, datasets cluster by target and not by treatment condition, further showing that treatment has little impact on results (Fig 4E, S12 Fig). Differential binding profiles were generated for groups of H3K4me3 versus RNA Pol II datasets separated by number of nuclei used, cell treatment, and combined for all datasets for the same target (Fig 4F). When groups are separated by these technical modifications, profiles of differential genes remain unchanged. These together suggest that any differences that exist between groups do not have a significant effect on the overall results. Differential binding profiles were generated for groups of H3K4me versus RNA Pol II datasets separated by number of nuclei used, cell treatment, and combined for all datasets for the same target (Fig 4F). When groups are separated for these technical modifications, profiles of differential genes remain unchanged. These together suggest that any differences that exist between groups do not have a significant effect on the overall results.

## Discussion

This study adapts the original CUT&RUN protocol for cultured primary B cells, which are a difficult cell type to work with, and it evaluates the reproducibility and robustness of a modified CUT&RUN. This optimized CUT&RUN protocol ensures the stability of samples, removes potential cytosolic contaminants, and is flexible enough to be used with a varying amount starting material or after freeze-thaw of samples. We have included major alterations to the original protocol, including very light fixation of cells to prevent sample loss, as well as significantly longer incubation times at critical steps, particularly during the MNase digestion. With the extended time for MNase digestion, it may be of concern that excess cleavage of chromatin can occur and decrease sensitivity of the technique. This protocol combats this issue by performing the digestion step under wet ice to ensure the reaction stays as cold as possible to keep the enzyme localized to its target. For H3K4me3 experiments from original and optimized CUT&RUN techniques, FRiP scores were similar (S4 Table), datasets clustered by PCA and in a binding affinity matrix (Fig 2D-E), and traces at individual genes appear similar (Fig 2C), supporting that the extended incubation does not negatively impact the quality of the results.

Fixation was necessary to consistently keep cultured primary B cells and their nuclei intact for the duration of the experiment, but it comes with potential complications including epitope masking, cleavage not completely freeing DNA fragments, and difficulty in data interpretation to name a few [33–37]. While this protocol does utilize fixation, it does so at a concentration (0.1%) and time (1 min) much lower than is typically used in ChIP-seq (1% formaldehyde for 10–15+ min) [38–42]. We show here that increasing the time of fixation and/or concentration of fixative has adverse consequences on library yield and results in more variation across samples, highlighting the importance of fixing the sample as lightly as possible for the factor of choice (here, RNA Pol II).

Although we have not tested samples of less than 100k nuclei here, it is likely that quality data can be obtained with less sample for strong antibody targets, as evidenced by the clean and robust signals reported here. While ChIP-seq requires cellular samples on the scale of millions, CUT&RUN can accomplish equal or better results with 200 times less sample – which is advantageous and even necessary when studying very limited and difficult cell types after fluorescence-activated cell sorting, such as germinal center B cells. A single immunized mouse may only give around 100k-500k total germinal center B cells, severely limiting the number of targets that can be probed and number of repeats when using ChIP-seq. With this modified CUT&RUN, more quality data can be obtained from far less sample, allowing a more in-depth analysis of targets of interest and the ability to perform multiple repeats, leading to more certainty of results. This study includes 63 original datasets for the many comparisons performed – a number that is just not possible with ChIP-seq for many labs due to the amount of sample and sequencing depth required. Additionally, the ability to probe difficult to capture, high molecular weight, and non-histone/non-transcription factor targets, such as RNA Pol II, with precision and reproducibility is a valuable benefit on its own. Identified binding sites of the histone modification H3K4me3 are relatively consistent across the variable technique modifications we tested. In contrast, binding and location of RNA Pol II within gene bodies is transient due to the processive nature of the enzyme. We anticipate this protocol will work effectively for sequence-specific transcription factors with the outlined technical modifications, further expanding its applicability to more scientists though it has yet to be confirmed. Additionally, while we were able to observe consistent and expected signals for RNA Pol II, it is possible that for particularly large complexes that collection of total DNA and size selection for the fragmented DNA could prove useful [2]. Current and future work is aimed at utilizing this technique for study of these additional targets, as well as use of more specific and limited primary B cell subsets.

## Supporting information

S1 Protocol Step-by-step protocol. (PDF)

S2 Table Resources table. (XLSX)

S3 Text Buffer recipes. (DOCX)

S4 Table Dataset information and analysis. (XLSX)

S5 Fig Nuclei recovery is better with fixation of cells and increased spermidine. Freshly cultured primary B cells (top, left) were either fixed (top, right) or left unfixed. Nuclei were prepared from fixed or unfixed cells (middle row). 125k nuclei were bound to magnetic ConA beads in 50 µL for 30 min on ice (bottom row). After incubation, supernatant was removed and beads were resuspended in an equal volume, then nuclei were counted. Number of nuclei/the initial 125k nuclei = % recovery. All images were taken using an automated cell counter and all counts were made using trypan blue staining and a hemocytometer.
(TIF)

S6 Fig Strip-tube shaking apparatus design. A-C shows front (A), side-angled (B), and bottom (C) views of block used for shaking CUT&RUN samples overnight. D shows how the block was designed to fit over the model of thermomixer used. E is an image our the initial apparatus to demonstrate all that is needed is something to stably hold the strip-tubes angled for efficient mixing.
(TIF)

S7 Fig Comparison of all datasets processed without a control for CUT&RUN Optimized, Original, and ChIP-seq. A-C: Genomic heatmaps and profiles showing aligned sequences from all ChIP-seq (ChIP), standard CUT&RUN (Original), and B cell-modified CUT&RUN (Optimized) for H3K4me3 (A), IgG (B), and RNA Pol II (C)) individually (no control). Shown is signals +/- 3 kb upstream and downstream from TSSs B: Individual tracks for H3K4me3, IgG, and RNA Pol II at select genes from ChIP-seq, standard CUT&RUN (Original), and B cell-modified CUT&RUN (Optimized). Traces shown are not normalized but instead auto-scaled to display features. E-F: Affinity matrix comparing all binding sites from normalized replicates (C) or differential (FDR </= 0.05) sites between H3K4me3 and RNA Pol II (D) from ChIP-seq, standard CUT&RUN, and B cell-modified CUT&RUN experiments for H3K4me3 (n=3, 1, 7, respectively), IgG (n=1, 1, 7, respectfully), and RNA Pol II (n=1, 1, 7, respectively). Datasets for ChIP-seq, original, and B cell-optimized CUT&RUN are indicated by the prefixes CS-, OG-, and S-, respectively, and groups by target (H3K4me3, IgG, or RNA Pol II) are indicated by the colored bars above. G-H: PCA analysis of peaks from all normalized datasets. (G) or differential binding between H3K4me3 and RNA Pol II (H). The solid oval indicates the H3K4me3 datasets, the dashed oval indicates the RNA Pol II datasets, and the fine dotted oval indicates the IgG datasets. Differential sites are those with an FDR </= 0.05.
(TIF)

S8 Fig Irreproducibility discovery rate analysis comparing peaks identified from ChIP-seq, standard CUT&RUN, and B cell-optimized CUT&RUN datasets. Peaks were identified from samples for H3K4me3 (left) or RNA Pol II (right) using Macs2 with IgG samples used as control and the setting “–broad”. IDR analysis was performed using the settings “--use-nonoverlapping-peaks” and “--use-best-multisummit-IDR”. Comparisons are Optimized CUT&RUN (C&R) (top), original CUT&RUN (C&R) (middle), and ChIP-seq (bottom). Black indicates an IDR < 0.05 and axes are log(10) scores.
(TIF)

S9 Fig Stronger fixation results in lower quality and quantity of libraries. CUT&RUN was performed at 500k cells each sample under one of the four fixation conditions (light, medium-low, medium-high, heavy). Resulting DNA fragments were used to prepare libraries for sequencing. Library size and quantity was obtained using the Bioanalyzer High Sensitivity DNA kit (Agilent) and Qubit 1x dsDNA High sensitivity kit (Invitrogen) (respectively). Traces from the Bioanalyzer are shown while yield (calculated from size data from the Bioanalyzer and concentration from the Qubit) are displayed at the top right of each trace.
(TIF)

S10 Fig A: Genomic heatmaps and profiles for H3K4me3, IgG, and RNA Pol II +/-3 kb from TSSs of individual datasets (no control) for light (0.1% formaldehyde for 1 min), medium-low (0.1% formaldehyde for 10 min), medium-high (1% formaldehyde for 1 min), and heavy (1% formaldehyde for 10 min) fixation conditions. B: IVG tracks for the four fixation conditions at select B cell (*Cd19*), DNA repair/CSR genes (*Apex1*, *Pcna*), and non-B cell genes (*Cd4*, *Cd3e*). C-D: Affinity matrices comparing cross-correlation for all normalized (C) or differentially bound (D) sites for group 2 datasets under the four treatment conditions. E-F: PCA plots for group 2 datasets at all normalized (E) or differentially bound (F) sites. Differential sites are those with an FDR </= 0.05.
(TIF)

S11 Fig Tracks for all fresh and frozen samples at select genes. A and B: Tracks for fresh (n=4, green) and frozen (n=6, blue) samples at select genes for HK4me3 (A), IgG (B) and RNA Pol II (C). Sample IDs are listed to the left of each trace (see S4 Table) while number of nuclei used is to the right.
(TIF)

S12 Fig PCA of affinity data for differential peaks. A and B: Differentially bound sites with an FDR <0.05 between the H3K4me3 and RNA Pol II sample groups were used to generate a PCA plot to visualize differences across groups. Samples are colored by input (A) or treatment (B). H3K4me3 datasets are enclosed in a solid oval while RNA Pol II datasets are enclosed in a dashed oval.
(TIF)
