# Supplementary material for: Optimized CUT&RUN protocol for activated primary mouse B cells
Source: PLoS One. 2025 Apr 24;20(4):e0322139. doi: 10.1371/journal.pone.0322139 (PMC12021426; doi:10.1371/journal.pone.0322139)
Supplement: S1 Protocol — (PDF) [file pone.0322139.s001.pdf]

# CUT&RUN protocol for activated primary B cells

RESERVED DOI:
**10.17504/protocols.io.3byl491y2go5/v1**

Stormy E. Ruiz[1]

[1]Laboratory of Molecular Biology and Immunology, National Institute on Aging, National Institutes of Health

Stormy E Ruiz
National Institute on Aging

**Protocol Info:** Stormy E. Ruiz . CUT&RUN protocol for activated primary B cells. **protocols.io** **https://protocols.io/view/cut-amp-run-protocol-for-activated-primary-b-cells-djkr4kv6**

**Created:** August 12, 2024

**Last Modified:** November 11, 2024

**Protocol Integer ID:** 105841

**Keywords:** CUT & RUN, Cleavage Under Targets Release Using Nuclease, B cells, Primary B cells

**Funders Acknowledgement:**
**Intramural Research Program at the National Institutes of Health, National Institute on Aging**
**Grant ID: AG000714**

## Abstract

CUT&RUN is a useful tool for the study of interactions between proteins and chromatin in limited samples. However, this technique was originally designed using cell lines and for targeting histones. Activated primary B cells are subject to degradation using the original methodology, so the protocol here was optimized for use on these cells. Further, this method was adapted and validated for non-histone proteins, such as the RNA polymerase II complex. This protocol is suitable for use on subsets of primary B cells and to target non-histone protein targets and post-translational modifications.

## Guidelines

- If you are stimulating cells fresh for CUT&RUN or want to prepare stimulated B cells for a later date, proceed to section titled B cell isolation.
- If you will be using previously frozen samples, proceed to section titled Thawing frozen samples.

# Materials

**Buffers:**

Make all buffers fresh on day 1 of the CUT&RUN experiment and keep all at 4°C or on ice at all times – especially once spermidine is added.

> **Note**
>
> Prepare aliquots of 1 M spermidine on ice and freeze. Thaw fresh aliquots on ice each time it is needed and discard remainer of tube after thaw.

**Pre-Nuclear Isolation Buffer:**

- 20 mM HEPES, pH 7.9
- 10 mM KCl
- 1 mM $MnCl_2$
- 0.1% Triton X-100
- 20% glycerol

Filter sterilize. Can be stored for up to 6 months at 4°C

**Nuclear Isolation Buffer:**

- Pre-Nuclear Isolation Buffer
- cOmplete EDTA-free Protease inhibitor
- PhosSTOP phosphatase inhibitor (if needed)
- 1 mM Spermidine

**Pre-XLS Wash Buffer:**

- Pre-Wash Buffer (20 mM HEPES-KOH pH 7.9, 150 mM NaCl) (in EpiCypher CUTANA CUT&RUN/ChIC kit)
- 1% Triton X-100
- 0.05% SDS
- Filter sterilize

**XLS-Wash Buffer:**

- Pre-XLS Wash Buffer
- cOmplete EDTA-free Protease inhibitor
- PhosSTOP phosphatase inhibitor (if needed)
- 1 mM Spermidine

**XLS-Antibody Binding Buffer:**

- XLS-Wash Buffer
- 2 mM EDTA

**Materials and Equipment:**

| A | B | C |
|---|---|---|
| **REAGENT or RESOURCE** | **SOURCE** | **IDENTIFIER** |
| **Antibodies** | | |
| H3K4me3 Antibody, SNAP-Certified for CUT&RUN and CUT&Tag | EpiCypher | Cat#13-0060 |
| CUTANA IgG Negative Control Antibody for CUT&RUN and CUT&Tag | EpiCypher | Cat#13-0042 |
| Anti-CD40 (mouse) mAb clone FGK45 | Enzo Life Sciences | Cat#ALX-805-046PF-C500; RRID: AB_10997445 |
| **Chemicals, peptides, and recombinant proteins** | | |
| RPMI Medium 1640 | Gibco | Cat#11875-101 |
| Fetal Bovine Serum, Heat Inactivated | Gibco | Cat#10-082-147 |
| GlutaMAX Supplement | Gibco | Cat#35050061 |
| Penicillin-Streptomycin (5,000 U/mL) | Gibco | Cat#15070063 |
| Betamercaptoethanol | Sigma Life Science | Cat#M3148-25ML, CAS: 60-24-2 |
| Lipopolysaccharide | Millipore Sigma | Cat#L2630-10MG |
| Recombinant mouse IL-4 | R&D Systems | Cat#404-ML/CF |
| ACK Lysing Buffer | Quality Biological | Cat#118-156-101 |
| Dimethylsulfoxide (DMSO) | Invitrogen | Cat#D12345; CAS: 67-68-5 |
| Dulbecco’s PBS (DPBS) | Gibco | Cat#14190-144 |
| Sodium Dodecyl Sulfate (SDS), 20% w/v | Quality Biological | Cat#351-066-721; CAS: 151-21-3 |
| Triton X-100 | Fisher Scientific | Cat#BP151-100, CAS: 9002-93-1 |
| cOmplete protease inhibitor, EDTA-free | Roche | Cat#11873580001 |
| HyPure Molecular Biology Grade Water | Cytiva | Cat#SH30538.03; CAS: 7732-18-5 |
| Manganese Chloride | Fisher Scientific | Cat#M87-100; CAS: 13446-34-9 |
| Proteinase K | Ambion | Cat#AM2548 |
| Spermidine | Acros Organics | Cat#132740010; CAS: 124-20-9 |
| HEPES | Sigma Life Science | Cat#H3375-250G; CAS: 7365-45-9 |
| Glycerol, 50% v/v | Ricca Chemical | Cat#3290-32; CAS: 56-81-5 |
| Formaldehyde, 37% by weight | Fisher Scientific | Cat#BP531-25; CAS: 50-00-0 |
| Glycine | Fisher Scientific | Cat#BP381-1; CAS: 56-40-6 |
| Trypan Blue | Invitrogen | Cat#T10282 |
| **Critical commercial assays** | | |
| CUTANA ChIC/CUT&RUN Kit v1-4 | EpiCypher | Cat#14-1048 |

| A | B | C |
|---|---|---|
| CUTANA CUT&RUN Library Prep Kit Primer Set 1 and Set 2 | EpiCypher | Cat#14-1001; Cat#14-1002 |
| Dead Cell Removal Kit | Miltenyi Biotec | Cat#130-090-101 |
| CD23 MicroBeads, mouse | Miltenyi Biotec | Cat#130-098-784 |
| High Sensitivity DNA Reagents Kit for Bioanalyzer 2100 | Agilent Technologies | Cat#5067-4626 |
| Qubit 1x dsDNA HS Assay Kit | Invitrogen | Cat#Q33231 |
| NovaSeq 6000 SP Reagent Kit v1.5 (200 cycles) | Illumina | Cat#20040719 |
| MiniSeq Mid Output Kit (300-cycles) | Illumina | Cat#FC-420-1004 |
| SPRIselect | Beckman Coulter | Cat#B23318 |
| **Software and algorithms** | | |
| BCL convert | Illumina | https://support.illumina.com/sequencing/sequencing_software/bcl-convert.html |
| TrimGalore | DOI 10.5281/zenodo.5127898 | https://github.com/FelixKrueger/TrimGalore |
| bowtie2 | Langmead and Salzberg | http://bowtie-bio.sourceforge.net/bowtie2/index.shtml |
| SAMtools | Li, et al. | http://samtools.sourceforge.net/ |
| MACS | Zhang, et.al. | https://macs3-project.github.io/MACS/ |
| IDR | Li, et al. | https://github.com/nboley/idr |
| Integrated Genomics Viewer (IGV) | Robinson, et. al. | https://igv.org/ |
| **Other** | | |
| Agilent Bioanalyzer 2100 | Agilent Technologies | https://www.agilent.com/en/product/automated-electrophoresis/bioanalyzer-systems/bioanalyzer-instrument/2100-bioanalyzer-instrument-228250 |
| Qubit 4 fluorometer | Invitrogen | Cat#Q33238 |
| MiniSeq | Illumina | Cat#SY-420-1001 |
| NovaSeq 6000 | Illumina | https://www.illumina.com/systems/sequencing-platforms/novaseq.html |
| Veriti Thermal Cycler, 96-Well | Applied Biosystems | Cat#4375305 |
| Strip-tube shaking block | Custom made in-house | N/A |
| Mr. Frosty freezing container | ThermoFisher Scientific | Cat#5100-0001 |
| Cell Strainer 70 µm Nylon | Falcon | Cat#352350 |
| LS Columns | Miltenyi Biotec | Cat#130-042-401 |
| MidiMACS Separator | Miltenyi Biotec | Cat#130-042-302 |
| EpiCypher 8-strip PCR tubes | Epicypher | Cat#10-0009 |
| 10X magnetic separator | 10x Genomics | Cat#120250 |

H3K4me3 Antibody, SNAP-Certified™ for CUT&RUN and CUT&Tag **EpiCypher Catalog #**13-0060

- CUTANA™ IgG Negative Control Antibody for CUT&RUN and CUT&Tag **EpiCypher Catalog #**13-0042
- CD40 (mouse) monoclonal antibody (FGK45) **Enzo Life Sciences Catalog #**ALX-805-046PF-C500
- RPMI 1640 Medium **Thermo Fisher Catalog #**11875101
- Heat-Inactivated FBS **Gibco - Thermo Fischer Catalog #**10-082-147
- GlutaMAX™ Supplement **Gibco - Thermo Fisher Catalog #**35050061
- Penicillin-Streptomycin (5,000 U/mL) **Thermo Fisher Catalog #**15070063
- b-mercaptoethanol **Merck MilliporeSigma (Sigma-Aldrich) Catalog #**M3148-25ML
- Lipopolysaccharides **Merck MilliporeSigma (Sigma-Aldrich) Catalog #**L2630-10MG
- Recombinant Mouse IL-4 Protein **R&D Systems Catalog #**404-ML
- ACK Lysing Buffer **Quality Biological Catalog #**118-156-101
- DMSO, Anhydrous **Thermo Fisher Catalog #**D12345
- Dulbeccos Phosphate Buffered Solution [DPBS] **Gibco - Thermo Fisher Catalog #**14190-144
- SDS (20%) **Quality Biological Catalog #**351-066-721
- Triton X-100 **Fisher Scientific Catalog #**BP151-100
- cOmplete™ EDTA-free Protease Inhibitor Cocktail **Roche Catalog #**11873580001
- Molecular Biology Grade Water **Cytiva Catalog #**SH30538.03
- Manganese Chloride **Fisher Scientific Catalog #**M87-100
- Proteinase K **Thermo Fisher Scientific Catalog #**AM2548
- Spermidine, 99% **Acros Organics Catalog #**132740010
- HEPES **Merck MilliporeSigma (Sigma-Aldrich) Catalog #**H3375
- Glycerin (Glycerol), 50% (v/v) **Ricca Chemical Company Catalog #**3290-32
- Formaldehyde (37% by Weight/Molecular Biology), Fisher BioReagents **Fisher Scientific Catalog #**BP531-25
- Glycine (White Crystals or Crystalline Powder) **Fisher Scientific Catalog #**BP381-1
- Trypan Blue **Invitrogen - Thermo Fisher Catalog #**T10282
- Cutana ChIC/CUT&RUN Kit **EpiCypher Catalog #**14-1048
- CUTANA CUT&RUN Library Prep Kit Primer Set 1 and Set 2 **EpiCypher Catalog #**14-1001 ; 14-1002
- Dead Cell Removal Kit **Miltenyi Biotec Catalog #**130-090-101
- CD23 MicroBeads, mouse **Miltenyi Biotec Catalog #**130-098-784
- Bioanalyzer High Sensitivity DNA Kit **Agilent Technologies Catalog #**5067-4626
- Qubit™ 1X dsDNA HS Assay Kit **Invitrogen - Thermo Fisher Catalog #**Q33231
- NovaSeq 6000 SP Reagent Kit v1.5 (200 cycles) **Illumina, Inc. Catalog #**20040719
- MiniSeq Mid Output Kit (300-cycles) **Illumina, Inc. Catalog #**FC-420-1004

- SPRIselect **Beckman Coulter** **Catalog #**B23318
- Qubit 4 Fluorometer **Thermo Fisher Scientific** **Catalog #**Q33238
- Illumina MiniSeq High Output Reagent Kit **Illumina, Inc.** **Catalog #**FC-420-1001
- Veriti™ 96-Well Fast Thermal Cycler **Thermo Fisher** **Catalog #**4375305
- Mr. Frosty™ Freezing Container **Thermo Fisher Scientific** **Catalog #**5100-0001
- Cell strainer 70um filter **Falcon** **Catalog #**352350
- LS Columns **Miltenyi Biotec** **Catalog #**130-042-401
- MidiMACS Separator **Miltenyi Biotec** **Catalog #**130-042-302
- CUTANA™ CUT&RUN 8-strip 0.2 mL Tubes **EpiCypher** **Catalog #**10-0009
- 10x Magnetic Separator **10x Genomics** **Catalog #**120250/ 230003

## Safety warnings

All standard institutional precautions and procedures should be taken when working with and disposing of biological samples and chemical reagents.

## Ethics statement

The procedure for use of animals for this experiment must be approved by your institution's Animal Care and Use Committee (ACUC) or equivalent ethics committee(s) PRIOR to conducting any experiments.

## Before start

**Before starting the protocol, ensure that you have:**

New aliquot(s) of frozen spermidine that has never been thawed.

- Spermidine that has been through multiple freeze-thaw cycles and/or has not been exclusively on ice when thawed may not work, resulting in total sample loss.
- I prepared 1 mL of [M] 1 Molarity (M) spermidine and On ice, aliquoted 10 µL each into PCR tubes which were then immediately frozen. I take out tubes as needed and thaw On ice.

Prepared the XLS-version of buffers for fixed samples. See Materials tab for complete recipes.

EDTA-free protease and/or phosphatase inhibitors.

- The MNase cleavage is calcium-dependent, thus EDTA will inhibit.

- A strong magnetic separator. Magnets used for Dynabeads may be insufficient to effectively pull-down beads. We use the 10X Genomics 10X Magnetic Separator HT (part no: 1000394).
- If you are unsure your magnet will work, test a small amount of Concanavalin A beads diluted in Bead Activation Buffer and try pipetting off the buffer and washing the beads with more Bead Activation buffer ( 10 µL buffer to 1 µL beads). If you keep pulling up beads upon aspiration or the beads are not being pulled to the magnet within a minute or two, you likely need a stronger magnet.

High-quality sample. This is critical, because if the cells are not well-stimulated and not prepared quickly and correctly, the nuclei will disintegrate when you mix with the ConA beads.

A brightfield microscope. You will need to visually inspect nuclei using trypan blue and a hemocytometer to check for integrity, so an automated cell counter may not be sufficient.

Filter pipet tips. Since we want to minimize any cross contamination across samples, the use of filter tips is highly recommended for all steps involving transfer of sample or reagents (i.e., primers, buffer for resuspending beads, etc.) Filter tips are not necessary for ConA bead washing steps during CUT&RUN, but change tips after each wash/disposal of buffer. Similarly, filter tips are not necessary for washes of SPRI beads as the washes are in ethanol.

## B cell Isolation and Stimulation: B cell Isolation

40m

1 Sacrifice mouse and remove spleen in tissue culture hood.

> **Note**
>
> All following steps in this section must be under sterile conditions.

2 Crush spleen through a 70-micron filter with MACS media to make a single cell suspension.

3 Centrifuge cells at 600 x g, Room temperature, 00:05:00 and remove supernatant. 5m

4 Resuspend cell pellet in 1 mL MACS media.

5 Add 5 mL ACK lysis buffer to remove red blood cells.

6 Centrifuge cells at 600 x g, Room temperature, 00:05:00 and remove supernatant. 5m

7 Resuspend cell pellet in 1 mL MACS media.

8 Count cells.

9 Add αCD23 magnetic beads according to number of total cells ( 100 µL per 100 million cells).

10 Chill sample at 4 °C for 00:15:00 - 00:20:00 . 20m

11 About 00:05:00 before end of incubation in go to step #10 , set up the LS column in the magnetic holder and wash with 7 mL MACS media. Discard flow-through. 5m

12 Pipet cells+beads directly onto the top of the column.

13 Once all sample is in the column, add 7 mL MACS media to wash. Discard flow-through.

14 Remove column from magnet and place into a 15-mL Falcon tube for collection.

15 Add 7 mL MACS media. Let half of the volume flow through by gravity, and then insert the plunger and firmly but gently, push the remaining sample through the column.

**Expected result**

This flow through is your purified follicular B cells.

16 Centrifuge cells at 600 x g, Room temperature, 00:05:00 and remove supernatant. 5m

17 Resuspend cells in 1 mL of MACS media or B cell media (see next section) and count cells.

## B cell Isolation and Stimulation: B cell stimulation

2d

18

**Note**

- A starting recommendation is to prepare more cells than you think you need, as it is better to have more sample to work with in case of technical issues.
- Plan for 2-3x expansion of cells with stimulation.
- You will lose cells if you freeze/thaw cells (about 40-80% yield) and again in the nuclei isolation step (about 60-90% yield).

**Note**

All following steps in this section must be under sterile conditions.

19 Prepare the B cell media:

Using pre-warmed RPMI 1640 as the starting buffer, supplement it with:

- 10% FBS
- 1% Glutamax
- 1% Pen/Strep
- [M] 53 micromolar (µM) beta-mercaptoethanol

Sterile filter.

**Note**

Media must be prepared same day as use for best results.

20 Add 250,000 cells per mL of media for stimulation.

21 Add 2 µg/ml αCD40 antibody and 10 ng/ml IL-4.

22 Aliquot cells into culture plate(s) and/or flask(s).

22.1 Aliquot 1 mL - 2 mL of culture into a separate plate for day 3 analysis of CSR to make sure your stimulation was successful. Instead of removing plate at 48 hours, remove at 72 hours and then stain for viability, IgG1, and IgM and perform flow cytometry analysis.

23 Incubate cultures at 5% $CO_2$ and 37 °C for 48:00:00 . 2d

## B cell Isolation and Stimulation: Sample collection (directly from culture) 10m

24 Remove culture from incubator and transfer into a Falcon tube.

25 Centrifuge at 600 x g, Room temperature, 00:05:00 and discard supernatant. 5m

26 Resuspend cell pellet in 1 mL of Room temperature DPBS and then dilute up to 10 mL .

27 Count cells.

28 Centrifuge cells once more at 600 x g, Room temperature, 00:05:00 . Discard supernatant. 5m

28.1 If using fresh cells, proceed directly to Nuclei preparation.

28.2 If freezing cells, resuspend pellet with 100% FBS and add DMSO to a final concentration of 10% v/v and aliquot into pre-labeled cryo-safe vials.

- Immediately place in Mr. Frosty slow freezer with isopropanol and place at -80 °C . Once frozen, you can remove tubes from Mr. Frosty and place in a standard freezer box.

**Note**

We recommend 5-10 million cells per mL, 1 mL per vial.

## B cell Isolation and Stimulation: Thawing frozen samples 10m

29 Remove cryo-safe vial(s) from -80 °C and immediately place in 37 °C water bath/incubator/heat block to quick thaw.

30 Once thawed, centrifuge at 600 x g, Room temperature, 00:05:00 and carefully pipet off supernatant, being careful to not disturb the pellet. 5m

**Note**

If your centrifuge does not fit cryovials, try placing the whole cryovial into a 14 mL Falcon tube(or equivalent) and then centrifuging. Use forceps to gently pull out the tube and aspirate off supernatant.

31 Resuspend pellet with 1 mL DPBS and transfer to 15 mL Falcon tube.

32 Add DPBS up to 10 mL .

33 Centrifuge at 600 x g, Room temperature, 00:05:00 and discard supernatant. (1/3) 5m

34 Resuspend pellet with 1 mL DPBS and take a small aliquot to count cells using Trypan Blue.

35 Add DPBS up to 10 mL .

36 Centrifuge at 600 x g, Room temperature, 00:05:00 and discard supernatant. (2/3) 5m

37 Resuspend pellet with 1 mL DPBS. Then add DPBS up to 10 mL .

38 Centrifuge at 600 x g, Room temperature, 00:05:00 and discard supernatant. (3/3) 5m

## Sample Preparation: Nuclei preparation 51m

39

**Note**

- It is important that sample preparation be timely with minimal time between steps. Make sure all reagents are prepared before proceeding. See supplemental file for buffer recipes.
- Fixing of cells and then nuclei preparation gives higher-quality sample than fixation of nuclei.
- Do not freeze nuclei – recovery and quality was extremely poor across multiple methods we tested.

Resuspend pellet with 100 µL of dead cell removal magnetic beads (or more if you have more than 10 million cells – refer to kit instructions).

40 Incubate at Room temperature for 00:15:00 . 15m

41 About 00:05:00 before incubation ends in go to step #40 , set up an LS column in the magnetic holder and wash with 3 mL DPBS. Discard flow-through. 5m

42 Place a 15 mL Falcon tube under the LS column (while on the magnet).

Note

You are **collecting flow-through** so this is critical.

43 Add DPBS to the sample for a final volume of 500 µL , and apply to the column.

44 Once all sample is in the column, add 6 mL - 7 mL DPBS and let it flow through. (1/2)

45 Add 6 mL - 7 mL DPBS and let it flow through. (2/2)

Note

Your total volume should be 12.5 mL - 14.5 mL .

46 Count and calculate total number of live cells.

47 Calculate how much formaldehyde is needed to add to your sample to get to a final concentration of 0.1% and have [M] 2.5 Molarity (M) glycine at hand for go to step #49 .

Note

For a 37% stock of formaldehyde you will use 2.7 µL/mL of your sample.

48 Add formaldehyde to 0.1% and immediately start a timer for 00:01:00 . Close the tube and continuously invert to mix (gently, not shaking). 1m

**Note**

It is recommended to fix one sample at a time due to the very short time frame for fixation. This ensures all samples are fixed for the correct length of time and evenly mixed with care.

49 After 60 seconds, immediately add glycine to a final concentration of [M] 125 millimolar (mM) (1:20 dilution) and mix well by gentle inversions.

50 Centrifuge sample at 600 x g, Room temperature, 00:05:00 and discard supernatant in appropriate and labeled waste container for fomaldehyde. 5m

**Note**

If working with a small number of cells (<1 million), very carefully aspirate off the supernatant instead of pouring off. This ensures minimal sample loss. This should be the case for all steps moving forward.

51 Resuspend pellet with 1 mL DPBS. (1/3)

51.1 Then, add DPBS up to 10 mL . (1/3)

52 Centrifuge at 600 x g, Room temperature, 00:05:00 and discard supernatant in appropriate and labeled waste container for fomaldehyde. (1/3) 5m

53 Resuspend pellet with 1 mL DPBS. (2/3)

53.1 Then, add DPBS up to 10 mL . (2/3)

54 Centrifuge at 600 x g, Room temperature, 00:05:00 and discard supernatant in appropriate and labeled waste container for fomaldehyde. (2/3) 5m

55 Resuspend pellet with 1 mL DPBS. (3/3)

55.1 Then, add DPBS up to 10 mL . (3/3)

56 Centrifuge at 600 x g, Room temperature, 00:05:00 and discard supernatant in appropriate and labeled waste container for fomaldehyde. (3/3)

5m

57 Add cold Nuclear Isolation Buffer at 1 mL per 5 million cells, minimum 1 mL.

**Note**

Do not pipet to mix – instead, gently vortex to reduce sample loss.

58 Incubate On ice for 00:10:00 .

10m

59 Centrifuge at 600 x g, 4°C, 00:05:00 and discard supernatant by careful aspiration. (1/2)

5m

60 Resuspend in 1 mL cold Wash-XLS buffer and then add up to 5 mL . Take a small aliquot, stain with trypan blue, and count nuclei while assessing quality. Sample will likely have scattered cell debris (appearance of dark blue fluff), but that is okay.

61 Centrifuge at 600 x g, 4°C, 00:05:00 and discard supernatant by careful aspiration. (2/2)

5m

62 Resuspend nuclei at 1-5 million nuclei per 1 mL in cold Wash-XLS buffer.

63 Again, take a small aliquot, stain with trypan blue and count nuclei for verification. There should be minimal to no cell debris left, but if it is still extensive, repeat wash step once more go to step #61 , resuspend nuclei go to step #62 , and recount. It is important that your sample is clear of debris to prevent clumping of samples during CUT&RUN.

64

**Note**

Prepared nuclei should always remain On ice or at 4 °C .

## Sample Preparation: Test nuclei stability with Concanavalin A beads (optional)

30m

65

**Note**

- The purpose of this section is to ensure sample will be able to withstand the CUT&RUN protocol, and it is important that this is done before beginning the experiment. By first preparing a small amount of beads and nuclei, there is no waste of ConA beads in case of sample failure.
- This section can be skipped when you determine your handling of samples to consistently yield stable nuclei.

66 Vortex Concanavalin A (ConA) beads to resuspend.

67 Take 3 µL and add to a tube (use 8-strip tubes from Epicypher supplied in kit).

68 Add 30 µL of Bead Activation buffer, mix, and place on magnetic separator to clear.

69 Remove supernatant and add 30 µL of Bead Activation Buffer.

70 Remove tube from magnet to mix by gentle pipetting and replace on magnetic separator to clear.

71 Remove supernatant and add 5 µL of Bead Activation Buffer.

72 Remove tube from magnet to mix by gentle pipetting.

73 Add 10 µL of prepared nuclei to beads, mix by gentle pipetting, and incubate On ice for 00:15:00 - 00:30:00 . 30m

74 Take an aliquot of nuclei+beads and stain with trypan blue. Observe sample under microscope. Nuclei should still be small, round, bright blue, and intact.

Note

- If you see an excessive amount of blue fluffy debris and abnormally-shaped nuclei, check that your buffers were made using the correct concentrations of reagents.
- You may attempt steps 64-70 again, but without knowing the cause of failure, it is unlikely the sample will be stable on the second attempt and will not withstand the CUT&RUN.
- There are many possible reasons this can happen – the most common tend to be poor stimulation of B cells, spermidine that is not fresh and/or not been kept On ice , taking too long to prepare samples/letting them sit around too long, and inadequate fixation, to name a few.
- Stop now and prepare new samples from the beginning.

## CUT&RUN: Binding nuclei to ConA beads

34m

75 Vortex beads to resuspend.

76 Take 11 µL of beads per CUT&RUN sample and transfer to a 1.5 mL microcentrifuge tube.

Note

This volume is the same regardless of how many nuclei you use. This ensures the magnetic pellet size is sufficient for pull down.

77 Add 100 µL of Bead Activation Buffer per sample and mix. (1/2)

78 Place tube on a magnetic separator and let clear (about 00:02:00 ). (1/2) 2m

79 Remove buffer and take tube off magnet. (1/2)

80 Add 100 µL of Bead Activation Buffer per sample and mix. (2/2)

81 Place tube on a magnetic separator and let clear (about 00:02:00 ). (2/2) 2m

82 Remove buffer and take tube off magnet. (2/2)

83 Resuspend beads in 11 µL Bead Activation Buffer per sample.

84 Dilute nuclei to desired concentration using Wash-XLS Buffer if desired. You will use 100 µL of nuclei each sample, so if you want to use 500,000 nuclei per sample, do not dilute. If you want to use 100,000 nuclei per sample, dilute nuclei 1 in 5, and so forth.

**Note**

If not using the whole sample, aliquot what will be used into a separate tube.

85 Add prepared ConA beads to the nuclei. Mix by gentle pipetting and incubate On ice for 00:30:00 . 30m

86 Take a small aliquot and stain using trypan blue. Check that nuclei are bright, round, and blue before proceeding.

87 Aliquot 110 µL of nuclei + beads each into 8-strip tubes.

**Note**

- Once the beads and nuclei have been bound and aliquoted into sample tubes, for all subsequent steps that involve mixing, we recommend tilting strip tubes horizontally and gently shaking back and forth or gentle vortexing and then quick spin.
- We do not recommend mixing by pipetting since the ConA beads will stick to most pipet tips, including low-retention tips, resulting in sample loss.

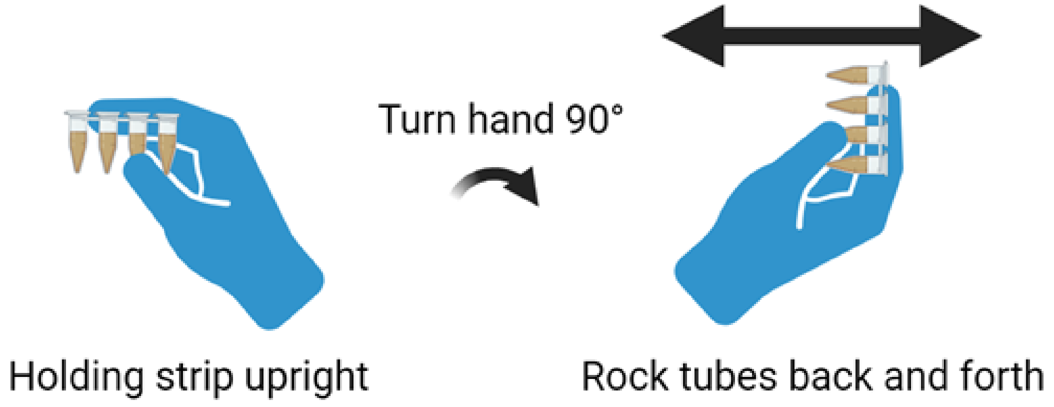


88 Place tubes with nuclei and ConA beads on magnetic separator and let clear (about 00:02:00 ). 2m

89 Remove supernatant and add 50 µL of Antibody Binding Buffer.

**Note**

- Do not mix by pipette: mix by tilting strip horizontally, gently shaking the strip back and forth, and then quick spin.
- A multi-channel pipet is highly recommended/helpful.

## CUT&RUN: Antibody Binding

8h

90 Add 1 µg of antibody to each sample for target of interest, including controls H3k4me3 and IgG.

**Note**

Some manufactures have recommended concentrations for their antibody clones for CUT&RUN – use those amounts if available instead of 1 µg.

91 Gently mix samples by shaking horizontally or gentle vortexing. Quick spin to collect samples to bottom of the tube.

92 Place samples on shaker or thermomixer (set to 800 rpm ) at a 30-45 degree angle from vertical kept at 4 °C Overnight . 8h

1. It is important for the tubes to be kept upright at an angle and with motion to ensure beads stay suspended. Do not rotate tubes – the reaction volume is small, and beads that get stuck on the cap will dry out, which will destroy your sample.
2. By the morning, most of the beads will have settled and that is normal – tilt the tubes on their side and gently mix by shaking and quick spin. Take note if beads are clumpy – this may be a sign that your nuclei have fallen apart.
   - If this happens, take 2 µL - 3 µL from one of your samples (take note of which) and add to 8 µL - 9 µL trypan blue and check under a microscope. Are the nuclei intact? Is there a lot of blue, fluffy debris? If there are no intact nuclei, the experiment has failed and you should start over.

## CUT&RUN: Binding and activation of pAG-MNase

2h 40m

93 Remove tubes from thermomixer. Gently agitate the tubes to resuspend beads and give a quick spin to collect all sample to the bottom of tube.

**Note**

Use of a metal tube block On ice is very helpful for keeping tubes stable and cold.

94 Place tubes on magnetic separator and let clear.

95 Remove supernatant and add 200 µL Wash-XLS Buffer while tubes are still on magnet. You do not need to resuspend the beads, just let the buffer gently wash over the beads. (1/2)

96 Remove supernatant and add 200 µL Wash-XLS Buffer while tubes are still on magnet. You do not need to resuspend the beads, just let the buffer gently wash over the beads. (2/2)

97 Remove supernatant and add 50 µL Wash-XLS Buffer. Gently shake horizontally or vortex to mix and quick spin to collect sample to the bottom of tube.

98 Add 2.5 µL pAG-MNase enzyme to each sample and gently shake horizontally or vortex to mix, and quick spin to collect sample to the bottom of tube.

99 Incubate tubes On ice for 00:30:00 . 30m

100 Place tubes on magnetic separator and let clear.

101 Remove supernatant and add 200 µL Wash-XLS Buffer while tubes are still on magnet. (1/2)

102 Remove supernatant and add 200 µL Wash-XLS Buffer while tubes are still on magnet. (2/2)

103 Remove supernatant and add 50 µL Wash-XLS Buffer. Gently shake or vortex to mix and quick spin to collect sample to the bottom of tube.

104 Incubate tubes on ice for 00:05:00 to ensure samples are cold. 5m

105 Add 1 µL [M] 100 millimolar (mM) Calcium Chloride to each sample. Mix and quickly centrifuge. Wrap samples in plastic wrap or place in a sealed bag, and incubate buried in wet ice for 02:00:00 . 2h

**Note**

Most complete digestion of fixed samples was found with a 2 hour digestion. Digestion at 30 minutes results in a larger proportion of multinucleosome fragments upon library preparation.

106 Prior to the end of the incubation, prepare the Stop Master Mix by adding 33 µL of Stop Buffer + 1 µL of *E. coli* Spike-in DNA per 500,000 nuclei for each sample and mix well.

**Note**

Spike-in should be approximately 1% of total reads. Scale amount of spike-in as needed for total amount of nuclei.

107 After 2 hours, add 33 µL of Stop Master Mix+spike-in to each sample. Very gently and briefly shake horizontally once to mix and quick spin to collect sample to the bottom of the tube.

108 Place samples in a thermocycler set to 37 °C for 00:10:00 . 10m

109 Quick spin samples and then place on magnetic separator to clear.

110 Collect supernatant and transfer to fresh 8-strip tubes.

## CUT&RUN: Post-CUT&RUN DNA purification 8h 23m

111 Add 1 µL Proteinase K and 1.6 µL of 5% SDS to each sample. Mix and quick spin.

112 Place samples in a thermocycler set to 55 °C and incubate Overnight to reverse-crosslink and digest protein. 8h

STEP CASE

For CUTANA Version 3 kit and older: 89 steps

113 Next day, transfer samples to 1.5 mL microcentrifuge tubes. Add 420 µL DNA Binding Buffer. Mix well and quick spin to collect sample.

114 Place DNA purification column in a 2 mL collection tube, and transfer each sample to a column.

115 Centrifuge at max speed ( 13000 x g - 16000 x g ) for 00:01:00 . Discard flow through. 1m

116 Add 200 µL DNA Wash Buffer to columns. (1/2)

117 Centrifuge at max speed ( 13000 x g - 16000 x g ) for 00:01:00 . Discard flow through. (1/2) 1m

118 Add 200 µL DNA Wash Buffer to columns. (2/2)

119 Centrifuge at max speed ( 13000 x g - 16000 x g ) for 00:01:00 . Discard flow through. (2/2) 1m

120 Centrifuge once more at max speed ( 13000 x g - 16000 x g ) for 00:01:00 to remove residual ethanol. Discard flow through. 1m

121 Transfer columns to clean 1.5 mL microcentrifuge tubes.

122 Add 12 µL DNA Binding Buffer to the center of each column without scratching the resin. Incubate at Room temperature for 00:05:00 . 5m

123 Centrifuge at max speed ( 13000 x g - 16000 x g ) for 00:01:00 . Discard columns. 1m

124 Before proceeding to library preparation, quantify yield of DNA from CUT&RUN experiments using a Qubit fluorometer with the 1x dsDNA High Sensitivity Assay Kit. Use 2 µL of each sample and 198 µL of 1x to analyze.

If possible, use this concentration to calculate the total yield of DNA (for the remaining 23 µL - 24 µL ).

**Note**

- Yields will likely be low and you may not get a concentration reading. Ideally, the positive H3K4me3 sample should have more DNA than the negative IgG control, but when using low numbers of nuclei, you may not get a concentration for most or any of the samples when using inputs lower than 250k. This is okay – quality libraries can still be prepared, and adjustments to the library preparation protocol have been added to accommodate this. If you consistently get no reading after many experiments with the same amount of starting input, it is fine to skip this step and proceed directly to library prep so as to not waste any sample.

## Library Preparation: End repair, adapter ligation, and U-excision

8h 23m

125 Thaw samples On ice , if frozen.

> **Note**
>
> - The library preparation should be performed in a PCR hood to minimize contamination.
> - Keep all reagents and mixes on ice unless thawing buffers (particularly End Repair buffer).
> - Use of a metal tube block On ice is very helpful for keeping tubes stable so they stay cold.

126 Thaw End Prep Buffer at Room temperature . Mix well and make sure all solids are dissolved, then place On ice . Thaw Adapter for Illumina On ice .

> **Note**
>
> Do not keep the adapter at Room temperature at any time. This will increase the amount of adapter dimer in the sample.

127 Transfer 5 ng of CUT&RUN fragments to new strip tubes. If you have less than 5 ng of total DNA (by Qubit), then use the entire 25 µL of sample.

128 Add 0.1x TE buffer to adjust sample volumes to 25 µL (if not already).

129 Prepare the End Repair Master mix as follows: Combine 4.2 µL End Prep Buffer and 1.8 µL of End Prep Enzyme per reaction. Mix by gentle vortexing and quick spin.

130 Add 5 µL of End Repair Master Mix to each sample. Mix by gentle vortexing and quick spin.

131 Place samples in a thermocycler and perform the following (with heated lid > 75 °C ):

- Step 1: 20 °C for 00:20:00
- Step 2: 65 °C for 00:30:00
- Hold at 4 °C

132 Remove tubes and quick spin to collect sample. Place tubes On ice .

**Note**

The following step using the thermocycler ( go to step #138 ) requires the lid to not be heated.

133 Prepare the Adapter Ligation Master Mix as follows: in a fresh tube, combine 16.5 µL of Ligation Mix and 0.5 µL Ligation enhancer each sample. Mix by gentle vortexing and quick spin, then place On ice .

- Do NOT add Adapter for Illumina to this mixture as the adapters will ligate together.

134 If first time using reagent, transfer Adapter for Illumina into a fresh tube On ice and add an equal volume of cold 0.1x TE buffer to make a 0.5x stock.

**Note**

Using less adapter reduces adapter dimer formation – especially when DNA yields are low.

135 Add 1.25 µL of diluted Adapter for Illumina to each sample.

136 Then, add 15.5 µL Ligation Master Mix to each sample.

137 Mix by gentle vortexing and quick spin.

138 Place samples in a thermocycler and incubate at 20 °C for 00:15:00 without heated lid.

139 Remove tubes from thermocycler, quick spin samples, and place in Room temperature rack.

140 Add 1 µL U-Excision Enzyme to each sample.

141 Mix by gentle vortexing and quick spin.

142 Place samples in a thermocycler and incubate at 37 °C for 00:15:00 with lid heated > 47 °C .

143 Remove tubes from thermocycler, quick spin samples.

**Note**

Samples can be stored at -20 °C at this point.

## Library Preparation: First DNA cleanup

8h 23m

144

**Note**

- A multichannel pipet and basins are very helpful here.
- If you have more than one 8-strip tubes and your magnetic separator holds only one strip, perform the cleanup one strip at a time to ensure protocol is accurately performed.

145 Before cleanup, prepare a fresh 85% mixture of ethanol in molecular biology-grade water. You will need ~ 500 µL per reaction for this first DNA cleanup.

146 Vortex SPRIselect beads ~ 00:00:30 to fully resuspend beads.

147 Add 47.75 µL SPRIselect beads to each sample. Mix by gentle vortexing and quick spin

148 Incubate samples at Room temperature for 00:05:00 .

149 Place tubes on magnetic separator and let clear. Remove supernatant.

150 Add 180 µL of 85% ethanol to samples (still on magnet), remove supernatant, and discard. (1/2)

151 Add 180 µL of 85% ethanol to samples (still on magnet), remove supernatant, and discard. (2/2)

152 Quick spin the tube strip and place back on magnetic separator. Remove any residual liquid.

153 Place the tubes on a rack at Room temperature and leaving lids open, let air dry for about 00:02:00 .

**Note**

Keep an eye on them – they should have little to no residual ethanol left and the beads will look slightly glossy, but do not let them completely dry (they will lighten in color and appear crumbly/cracked).

154 Add 12 µL of 0.1x TE buffer to each tube. Vortex to resuspend beads, quick spin, and incubate at Room temperature for 00:02:00 to release DNA fragments.

155 Place tubes on magnetic separator and let clear. Collect ~ 10.5 µL of supernatant and transfer to fresh 8-strip tubes, being careful to not pull up any beads even if there is a small volume of buffer left.

**Note**

Samples can be stored at -20 °C at this point.

## Library Preparation: Indexing PCR

8h 23m

156

**Note**

- Use of a metal tube block on ice is very helpful for keeping tubes stable and cold.
- Prior to first use, transfer 10 µL of each indexing primer to 8-strip tubes (in order for easy pipetting with multichannel). Add 40 µL of 0.1x TE buffer to each primer to make a 1 in 5 dilution. Be careful to ensure that there is no cross contamination between primers.

157 Identify what unique combination of indexing primers (i7 and i5) will be used for each sample and take note. The CUT&RUN Library Prep kit comes with a very helpful table on the Quick-Start card for recording which combinations have already been used.

158 Thaw indexing primers On ice . Quick spin if needed.

159 Add 1 µL of assigned and diluted i7 primer to each sample.

160 Add 1 µL of assigned and diluted i5 primer to each sample.

161 Add 12.5 µL of Hot Start 2x PCR Master Mix to each sample. Total volume should be 25 µL .

162 Mix by gentle vortexing and quick spin.

163 Place tubes in a thermocycler with the following PCR steps (with heated lid 105 °C ):

| A | B | C | D |
|---|---|---|---|
| Step 1 | 98°C | 45 seconds | |
| Step 2 | 98°C | 15 seconds | 16 cycles |
| | 60°C | 10 seconds | |
| Step 3 | 72°C | 1 minute | |
| Hold | 4°C | | |

PCR cycling conditions

164 Remove tubes from thermocycler and quick spin.

> **Note**
>
> Samples can be stored at -20 °C at this point.

## Library Preparation: Second DNA cleanup

8h 23m

165

> **Note**
>
> - This is a multi-step cleanup. You will perform a double-sided SPRIselect beads selection at 0.6-0.95x ratios. This helps remove any excessive adapter dimers and most multinucleosome fragments.
> - If you have more than one set of 8-strip tubes and your magnetic separator holds only one strip, I recommend performing the cleanup one strip at a time to ensure protocol is accurately performed.

166 Prepare fresh 85% ethanol in MB-grade water if needed. You will need about 900 µL - 1000 µL for each sample.

167 Add 25 µL of 0.1x TE to each tube. Total volume should now be 50 µL .

168 Vortex SPRIselect beads ~ 00:00:30 to fully resuspend beads.

169 Add 32.5 µL SPRIselect beads to each sample (equal to 0.6x ratio of beads to sample). Mix by gentle vortexing and quick spin.

170 Incubate samples at Room temperature for 00:03:00 .

171 Place tubes on magnetic separator and let clear. **Collect supernatant** and transfer it to fresh 8-strip tubes.

**Note**

Library is in the supernatant as it is excluded from the beads due to the low ratio. Do not throw the supernatant away!

172 Add 15 µL SPRIselect beads to each sample (equal to 0.35x, bringing total added to 0.95x). Mix by gentle vortexing and quick spin.

173 Incubate samples at Room temperature for 00:05:00 .

174 Place tubes on magnetic separator and let clear. Remove supernatant.

175 Add 180 µL of 85% ethanol to samples (still on magnet), remove supernatant, and discard. (1/2)

176 Add 180 µL of 85% ethanol to samples (still on magnet), remove supernatant, and discard. (2/2)

177 Quick spin the tube strip and place back on magnetic separator. Remove any residual liquid.

178 Place the tubes on a rack at Room temperature and leaving lids open, let air dry for about 00:00:30 - 00:01:00 .

**Note**

Keep an eye on them – they should have little to no residual ethanol left and the beads will look glossy, but do not let them completely dry (they will lighten in color and appear crumbly/cracked). They will dry very quickly due to the low volume of beads.

179 Add 31.5 µL of 0.1x TE buffer to each tube. Vortex to resuspend beads, quick spin, and incubate at Room temperature for 00:05:00 to release DNA fragments.

180 Place tubes on magnetic separator and let clear.

181 Collect 30 µL supernatant and transfer to fresh 8-strip tubes, being careful to not pull up any beads even if there is a small volume of buffer left.

**Note**

DNA can be stored at -20 °C until ready to perform sequencing.

## Sequencing: Library QC

8h 23m

182

**Note**

Before proceeding to sequencing, check libraries by both Qubit (for concentration) and Bioanalyzer (for fragment size distribution):

183 For Qubit, use 1 µL - 2 µL of library with 1x dsDNA high sensitivity reagent up to 200 µL .

184 For Bioanalyzer, use Agilent High Sensitivity DNA kit. Follow manufacturers protocol.

185 Using these results, calculate the molarity and/or moles of each library.

## Sequencing

8h 23m

186 Determine which sequencing kit you will use for your samples.

**Note**

For sequencing, paired end and minimum 50 bps is needed to map data. Depending on the target, 3-5 million reads may be enough, but if this is your first time running a target (especially non-histone), then plan to sequence 10-15 million reads each sample.

187 Pool libraries into equimolar ratios.

- Prepare an equimolar mixture of libraries for sequencing, calculated by sequence-able fragment sizes (~500 bps and under).
- If needed, perform one more SPRIselect bead purification on library pool to remove any remaining adapter dimer and/or multinucleosome fragments.
- Reanalyze using Qubit and Bioanalyzer

188 Sequence using an appropriate sequencing kit.

## Sequencing: Data Analysis

8h 23m

189 Copy raw data files from the sequencer to your computer.

**Note**

It's always a good idea to have backups of the data as well.

190 Using bcl2convert (Illumina), perform demultiplexing.

191 Using Trim Galore!, trim adapters off the reads.

- Options --paired --fastqc --fastqc_args "--nogroup"

192 Using bowtie2, align reads to the appropriate genome (mm39, hg38, etc.).

- Mode -x, -1 and -2 flags for paired sample files.

193 Using SAMtools, perform the function collate.

194 Using SAMtools, perform the function fixmate.

- Options -rm

195 Using SAMtools, perform the function sort.

- Options -@ 8 -T $TMPDIR

196 Using SAMtools, perform the function markdup.

- Options -r -T $TMPDIR

197 Using MACS2, perform the function callpeak.

- Options -t for sample, -c for IgG control, -B

198 Using Integrative Genome Browser, input your sequence (.fa) and annotated gene files (.gff or similar) for genome of choice.

199 Using Integrative Genome Browser, input -pileup.bdg peak files.

200 Data can now be visualized by chromosome and gene.

201 Additional analyses may be performed as appropriate, such as IDR, motif analysis, etc.

STEP CASE

For CUTANA Version 4 kit and newer: 90 steps

113 Prepare fresh 85% EtOH. You will need about 500 µL per sample but make more so that you can use a multichannel pipet and basin.

114 Mix SPRI beads well and add 120 µL to each sample. Mix tubes by gently shaking or vortexing and quick spin.

115 Incubate at Room temperature for 00:05:00 . 5m

116 Place strip on the magnetic separator and let clear.

117 Remove supernatant and wash with 180 µL of 85% EtOH. (1/2)

118 Remove supernatant and wash with 180 µL of 85% EtOH. (2/2)

119 Quick spin tubes and then reapply to magnet. Pipette off any remaining supernatant, being careful to not remove beads.

120 Let beads air dry – time depends on how much EtOH remains in the tubes and the amount of beads. The beads will take around 00:02:00 - 00:05:00 to become "dry". 7m

- You're looking for a matte texture and appearance. If the beads are glossy and smooth, they are too wet. If the beads are cracked and dry, they are too dry and add elution buffer immediately.

121 Add 26 µL of 0.1x TE buffer, mix resuspend beads by vortexing, and quick spin.

122 Incubate for 00:05:00 at Room temperature . 5m

123 Place tubes on magnetic separator and let clear.

124 Using a single channel pipet, collect 25 µL - 26 µL of elute from the beads and place into a new tube.

- Single channel is recommended because you will have more control over aspiration and avoiding the beads – you do not want any bead contamination.

**Note**

DNA can be stored at -20 °C until ready to perform library preparation. It is recommended to perform library prep of samples in bulk to minimize prep variation.

## Library Preparation: End repair, adapter ligation, and U-excision

1h 20m

125 Before proceeding to library preparation, quantify yield of DNA from CUT&RUN experiments using a Qubit fluorometer with the 1x dsDNA High Sensitivity Assay Kit. Use 2 µL of each sample and 198 µL of 1x to analyze.

If possible, use this concentration to calculate the total yield of DNA (for the remaining 23 µL - 24 µL ).

**Note**

- Yields will likely be low and you may not get a concentration reading. Ideally, the positive H3K4me3 sample should have more DNA than the negative IgG control, but when using low numbers of nuclei, you may not get a concentration for most or any of the samples when using inputs lower than 250k. This is okay – quality libraries can still be prepared, and adjustments to the library preparation protocol have been added to accommodate this. If you consistently get no reading after many experiments with the same amount of starting input, it is fine to skip this step and proceed directly to library prep so as to not waste any sample.

126 Thaw samples On ice , if frozen.

**Note**

- The library preparation should be performed in a PCR hood to minimize contamination.
- Keep all reagents and mixes on ice unless thawing buffers (particularly End Repair buffer).
- Use of a metal tube block On ice is very helpful for keeping tubes stable so they stay cold.

127 Thaw End Prep Buffer at Room temperature . Mix well and make sure all solids are dissolved, then place On ice . Thaw Adapter for Illumina On ice .

**Note**

Do not keep the adapter at Room temperature at any time. This will increase the amount of adapter dimer in the sample.

128 Transfer 5 ng of CUT&RUN fragments to new strip tubes. If you have less than 5 ng of total DNA (by Qubit), then use the entire 25 µL of sample.

129 Add 0.1x TE buffer to adjust sample volumes to 25 µL (if not already).

130 Prepare the End Repair Master mix as follows: Combine 4.2 µL End Prep Buffer and 1.8 µL of End Prep Enzyme per reaction. Mix by gentle vortexing and quick spin.

131 Add 5 µL of End Repair Master Mix to each sample. Mix by gentle vortexing and quick spin.

132 Place samples in a thermocycler and perform the following (with heated lid > 75 °C ): 50m

- Step 1: 20 °C for 00:20:00
- Step 2: 65 °C for 00:30:00
- Hold at 4 °C

133 Remove tubes and quick spin to collect sample. Place tubes On ice .

**Note**

The following step using the thermocycler ( go to step #139 ) requires the lid to not be heated.

134 Prepare the Adapter Ligation Master Mix as follows: in a fresh tube, combine 16.5 µL of Ligation Mix and 0.5 µL Ligation enhancer each sample. Mix by gentle vortexing and quick spin, then place On ice .

- Do NOT add Adapter for Illumina to this mixture as the adapters will ligate together.

135 If first time using reagent, transfer Adapter for Illumina into a fresh tube On ice and add an equal volume of cold 0.1x TE buffer to make a 0.5x stock.

**Note**

Using less adapter reduces adapter dimer formation – especially when DNA yields are low.

136 Add 1.25 µL of diluted Adapter for Illumina to each sample.

137 Then, add 15.5 µL Ligation Master Mix to each sample.

138 Mix by gentle vortexing and quick spin.

139 Place samples in a thermocycler and incubate at 20 °C for 00:15:00 without heated lid.

15m

140 Remove tubes from thermocycler, quick spin samples, and place in Room temperature rack.

141 Add 1 µL U-Excision Enzyme to each sample.

142 Mix by gentle vortexing and quick spin.

143 Place samples in a thermocycler and incubate at 37 °C for 00:15:00 with lid heated > 47 °C .

15m

144 Remove tubes from thermocycler, quick spin samples.

**Note**

Samples can be stored at -20 °C at this point.

## Library Preparation: First DNA cleanup

9m 30s

145

**Note**

- A multichannel pipet and basins are very helpful here.
- If you have more than one 8-strip tubes and your magnetic separator holds only one strip, perform the cleanup one strip at a time to ensure protocol is accurately performed.

146 Before cleanup, prepare a fresh 85% mixture of ethanol in molecular biology-grade water. You will need ~ 500 µL per reaction for this first DNA cleanup.

147 Vortex SPRIselect beads ~ 00:00:30 to fully resuspend beads. 30s

148 Add 47.75 µL SPRIselect beads to each sample. Mix by gentle vortexing and quick spin

149 Incubate samples at Room temperature for 00:05:00 . 5m

150 Place tubes on magnetic separator and let clear. Remove supernatant.

151 Add 180 µL of 85% ethanol to samples (still on magnet), remove supernatant, and discard. (1/2)

152 Add 180 µL of 85% ethanol to samples (still on magnet), remove supernatant, and discard. (2/2)

153 Quick spin the tube strip and place back on magnetic separator. Remove any residual liquid.

154 Place the tubes on a rack at Room temperature and leaving lids open, let air dry for about 00:02:00 . 2m

**Note**

Keep an eye on them – they should have little to no residual ethanol left and the beads will look slightly glossy, but do not let them completely dry (they will lighten in color and appear crumbly/cracked).

155 Add 12 µL of 0.1x TE buffer to each tube. Vortex to resuspend beads, quick spin, and incubate at Room temperature for 00:02:00 to release DNA fragments. 2m

156 Place tubes on magnetic separator and let clear. Collect ~ 10.5 µL of supernatant and transfer to fresh 8-strip tubes, being careful to not pull up any beads even if there is a small volume of buffer left.

Note

Samples can be stored at -20 °C at this point.

## Library Preparation: Indexing PCR

2m 10s

157

Note

- Use of a metal tube block on ice is very helpful for keeping tubes stable and cold.
- Prior to first use, transfer 10 µL of each indexing primer to 8-strip tubes (in order for easy pipetting with multichannel). Add 40 µL of 0.1x TE buffer to each primer to make a 1 in 5 dilution. Be careful to ensure that there is no cross contamination between primers.

158 Identify what unique combination of indexing primers (i7 and i5) will be used for each sample and take note. The CUT&RUN Library Prep kit comes with a very helpful table on the Quick-Start card for recording which combinations have already been used.

159 Thaw indexing primers On ice . Quick spin if needed.

160 Add 1 µL of assigned and diluted i7 primer to each sample.

161 Add 1 µL of assigned and diluted i5 primer to each sample.

162 Add 12.5 µL of Hot Start 2x PCR Master Mix to each sample. Total volume should be 25 µL .

163 Mix by gentle vortexing and quick spin.

164 Place tubes in a thermocycler with the following PCR steps (with heated lid 105 °C ):

| A | B | C | D |
|---|---|---|---|
| Step 1 | 98°C | 45 seconds | |
| Step 2 | 98°C | 15 seconds | 16 cycles |
| | 60°C | 10 seconds | |
| Step 3 | 72°C | 1 minute | |
| Hold | 4°C | | |

PCR cycling conditions

165 Remove tubes from thermocycler and quick spin.

**Note**

Samples can be stored at -20 °C at this point.

## Library Preparation: Second DNA cleanup

14m 30s

166

**Note**

- This is a multi-step cleanup. You will perform a double-sided SPRIselect beads selection at 0.6-0.95x ratios. This helps remove any excessive adapter dimers and most multinucleosome fragments.
- If you have more than one set of 8-strip tubes and your magnetic separator holds only one strip, I recommend performing the cleanup one strip at a time to ensure protocol is accurately performed.

167 Prepare fresh 85% ethanol in MB-grade water if needed. You will need about 900 µL - 1000 µL for each sample.

168 Add 25 µL of 0.1x TE to each tube. Total volume should now be 50 µL .

169 Vortex SPRIselect beads ~ 00:00:30 to fully resuspend beads. 30s

170 Add 32.5 µL SPRIselect beads to each sample (equal to 0.6x ratio of beads to sample). Mix by gentle vortexing and quick spin.

171 Incubate samples at Room temperature for 00:03:00 . 3m

172 Place tubes on magnetic separator and let clear. **Collect supernatant** and transfer it to fresh 8-strip tubes.

> **Note**
>
> Library is in the supernatant as it is excluded from the beads due to the low ratio. Do not throw the supernatant away!

173 Add 15 µL SPRIselect beads to each sample (equal to 0.35x, bringing total added to 0.95x). Mix by gentle vortexing and quick spin.

174 Incubate samples at Room temperature for 00:05:00 . 5m

175 Place tubes on magnetic separator and let clear. Remove supernatant.

176 Add 180 µL of 85% ethanol to samples (still on magnet), remove supernatant, and discard. (1/2)

177 Add 180 µL of 85% ethanol to samples (still on magnet), remove supernatant, and discard. (2/2)

178 Quick spin the tube strip and place back on magnetic separator. Remove any residual liquid.

179 Place the tubes on a rack at Room temperature and leaving lids open, let air dry for about 00:00:30 - 00:01:00 . 1m

Note

Keep an eye on them – they should have little to no residual ethanol left and the beads will look glossy, but do not let them completely dry (they will lighten in color and appear crumbly/cracked). They will dry very quickly due to the low volume of beads.

180 Add 31.5 µL of 0.1x TE buffer to each tube. Vortex to resuspend beads, quick spin, and incubate at Room temperature for 00:05:00 to release DNA fragments. 5m

181 Place tubes on magnetic separator and let clear.

182 Collect 30 µL supernatant and transfer to fresh 8-strip tubes, being careful to not pull up any beads even if there is a small volume of buffer left.

Note

DNA can be stored at -20 °C until ready to perform sequencing.

## Sequencing: Library QC

183

Note

Before proceeding to sequencing, check libraries by both Qubit (for concentration) and Bioanalyzer (for fragment size distribution):

184 For Qubit, use 1 µL - 2 µL of library with 1x dsDNA high sensitivity reagent up to 200 µL .

185 For Bioanalyzer, use Agilent High Sensitivity DNA kit. Follow manufacturers protocol.

186 Using these results, calculate the molarity and/or moles of each library.

## Sequencing

187 Determine which sequencing kit you will use for your samples.

> **Note**
>
> For sequencing, paired end and minimum 50 bps is needed to map data. Depending on the target, 3-5 million reads may be enough, but if this is your first time running a target (especially non-histone), then plan to sequence 10-15 million reads each sample.

188 Pool libraries into equimolar ratios.

- Prepare an equimolar mixture of libraries for sequencing, calculated by sequence-able fragment sizes (~500 bps and under).
- If needed, perform one more SPRIselect bead purification on library pool to remove any remaining adapter dimer and/or multinucleosome fragments.
- Reanalyze using Qubit and Bioanalyzer

189 Sequence using an appropriate sequencing kit.

## Sequencing: Data Analysis

190 Copy raw data files from the sequencer to your computer.

> **Note**
>
> It's always a good idea to have backups of the data as well.

191 Using bcl2convert (Illumina), perform demultiplexing.

192 Using Trim Galore!, trim adapters off the reads.

- Options --paired --fastqc --fastqc_args "--nogroup"

193 Using bowtie2, align reads to the appropriate genome (mm39, hg38, etc.).

- Mode -x, -1 and -2 flags for paired sample files.

194 Using SAMtools, perform the function collate.

195 Using SAMtools, perform the function fixmate.

- Options -rm

196 Using SAMtools, perform the function sort.

- Options -@ 8 -T $TMPDIR

197 Using SAMtools, perform the function markdup.

- Options -r -T $TMPDIR

198 Using MACS2, perform the function callpeak.

- Options -t for sample, -c for IgG control, -B

199 Using Integrative Genome Browser, input your sequence (.fa) and annotated gene files (.gff or similar) for genome of choice.

200 Using Integrative Genome Browser, input -pileup.bdg peak files.

201 Data can now be visualized by chromosome and gene.

202 Additional analyses may be performed as appropriate, such as IDR, motif analysis, etc.

# Protocol references

**Citations:**

This protocol has been adapted with Epicypher CUTANA ChIC/CUT&RUN (SKU: 14-1048) and CUTANA Library prep kits (SKU: 14-1001 & SKU: 14-1002) and protocols along the original protocols published by Skene and Henikoff.

1. Skene, P. & Steven Henikoff, S. An efficient targeted nuclease strategy for high-resolution mapping of DNA binding sites eLife 6:e21856 (2017). https://doi.org/10.7554/eLife.21856

2. Skene, P., Henikoff, J. & Henikoff, S. Targeted in situ genome-wide profiling with high efficiency for low cell numbers. Nat Protoc 13, 1006–1019 (2018). https://doi.org/10.1038/nprot.2018.015
