# Supplementary material for: Optimized CUT&RUN protocol for activated primary mouse B cells
Source: PLoS One. 2025 Apr 24;20(4):e0322139. doi: 10.1371/journal.pone.0322139 (PMC12021426; doi:10.1371/journal.pone.0322139)
Supplement: S3 Text — (DOCX) [file pone.0322139.s003.docx]

B Cell CUT&RUN Buffer Recipes

Make all buffers fresh on day 1 of the CUT&RUN experiment and keep all at 4°C or on ice at all times – especially once spermidine is added.

Note: Prepare aliquots of 1 M spermidine on ice and freeze. Thaw *fresh* aliquots on ice each time it is needed and discard remainer of tube after thaw.

Pre-Nuclear Extraction Buffer: 20 mM HEPES pH 7.9

10 mM KCl

1 mM MnCl_2_

0.1% Triton X-100

20% Glycerol

*Filter Sterilize, can be stored up to 6 months at 4°C*

Nuclear Extraction Buffer: Pre-Nuclear Extraction Buffer

cOmplete EDTA-free Protease inhibitor

PhosSTOP phosphatase inhibitor (if needed)

1 mM Spermidine

Pre-XLS Wash Buffer: Pre-Wash Buffer (20 mM HEPES-KOH pH 7.9, 150 mM NaCl) (in EpiCypher CUTANA CUT&RUN/ChIC kit)

1% Triton X-100

0.05% SDS

*Filter sterilize*

XLS-Wash Buffer: Pre-XLS Wash Buffer

cOmplete EDTA-free Protease inhibitor

PhosSTOP phosphatase inhibitor (if needed)

1 mM Spermidine

XLS-Antibody Binding Buffer: XLS-Wash Buffer

2 mM EDTA
