# Supplementary figures and images for: Optimized CUT&RUN protocol for activated primary mouse B cells
Source: PLoS One. 2025 Apr 24;20(4):e0322139. doi: 10.1371/journal.pone.0322139 (PMC12021426; doi:10.1371/journal.pone.0322139)

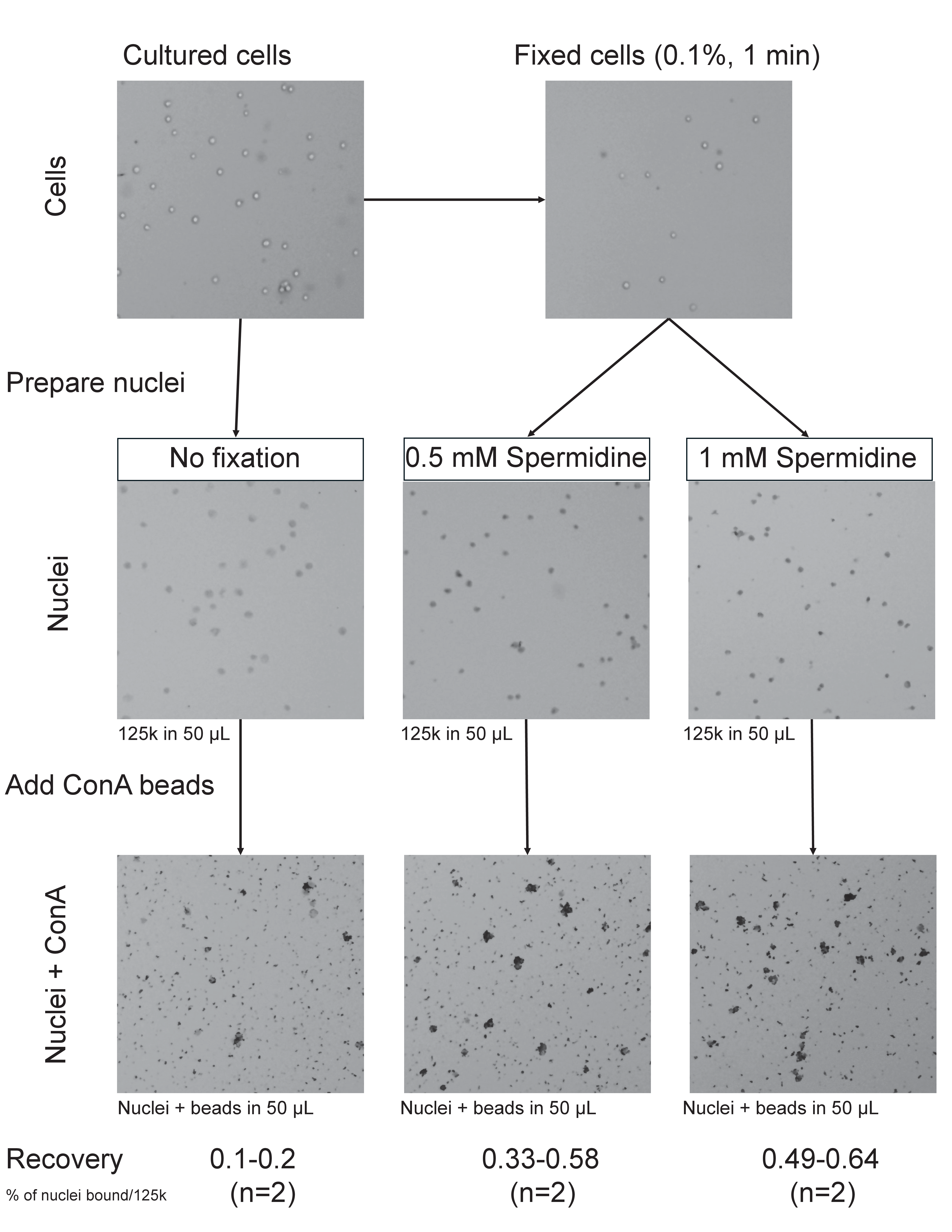

Supplement: S5 Fig — Freshly cultured primary B cells (top, left) were either fixed (top, right) or left unfixed. Nuclei were prepared from fixed or unfixed cells (middle row). 125k nuclei were bound to magnetic ConA beads in 50 µL for 30 min on ice (bottom row). After incubation, supernatant was removed and beads were resuspended in an equal volume, then nuclei were counted. Number of nuclei/the initial 125k nuclei = % recovery. All images were taken using an automated cell counter and all counts were made using trypan blue staining and a hemocytometer. (TIF) [file pone.0322139.s005.tif]

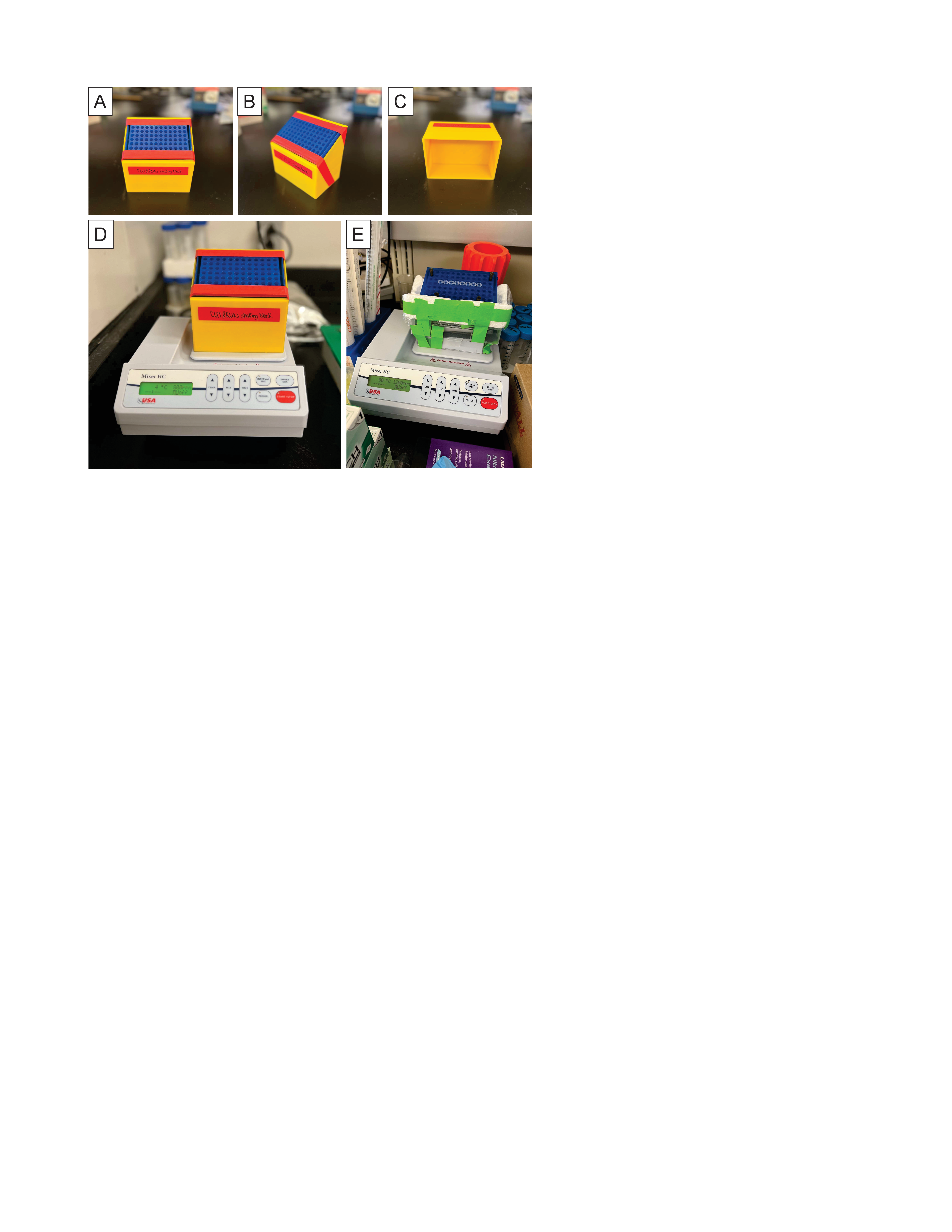

Supplement: S6 Fig — A-C shows front (A), side-angled (B), and bottom (C) views of block used for shaking CUT&RUN samples overnight. D shows how the block was designed to fit over the model of thermomixer used. E is an image our the initial apparatus to demonstrate all that is needed is something to stably hold the strip-tubes angled for efficient mixing. (TIF) [file pone.0322139.s006.tif]

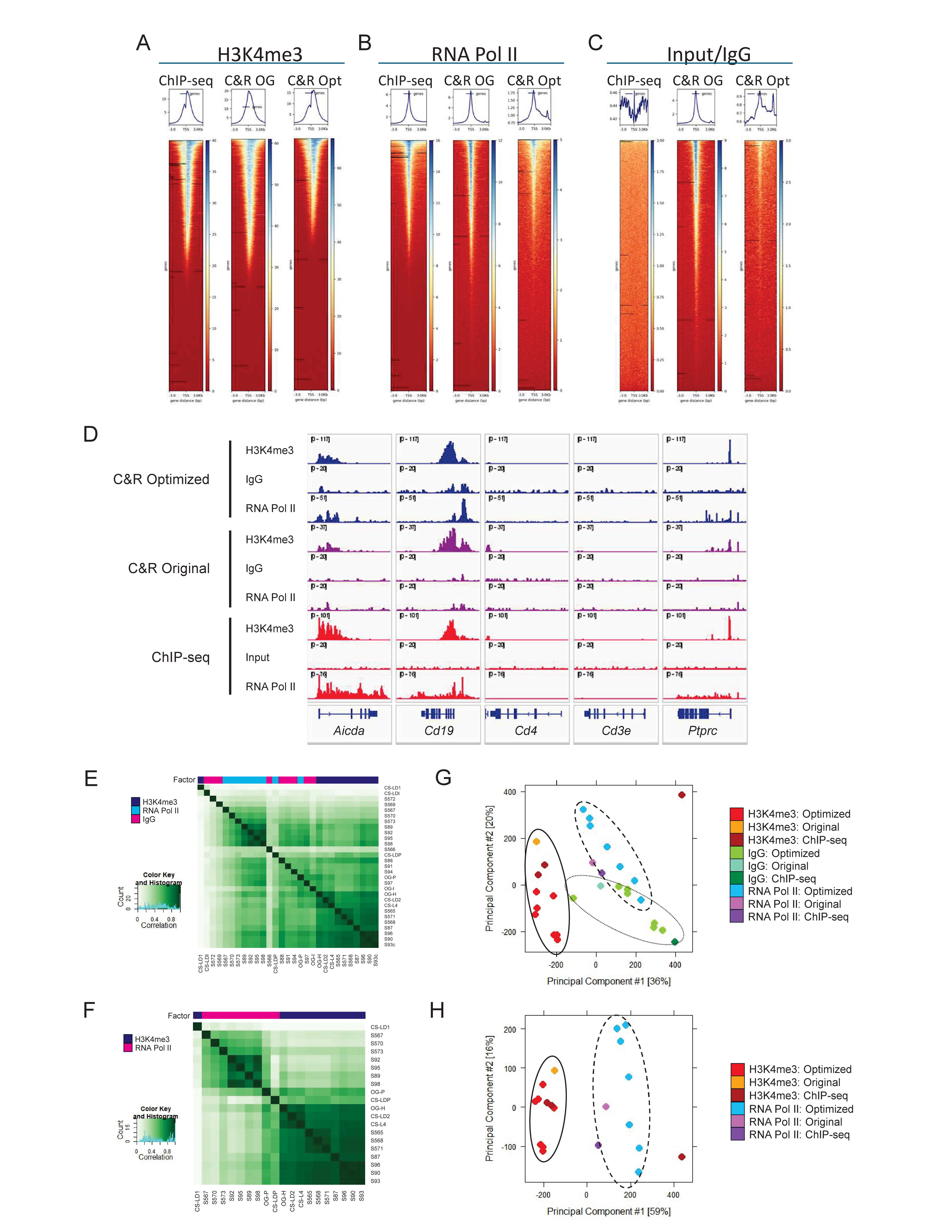

Supplement: S7 Fig — A-C: Genomic heatmaps and profiles showing aligned sequences from all ChIP-seq (ChIP), standard CUT&RUN (Original), and B cell-modified CUT&RUN (Optimized) for H3K4me3 (A), IgG (B), and RNA Pol II (C)) individually (no control). Shown is signals +/- 3 kb upstream and downstream from TSSs B: Individual tracks for H3K4me3, IgG, and RNA Pol II at select genes from ChIP-seq, standard CUT&RUN (Original), and B cell-modified CUT&RUN (Optimized). Traces shown are not normalized but instead auto-scaled to display features. E-F: Affinity matrix comparing all binding sites from normalized replicates (C) or differential (FDR </= 0.05) sites between H3K4me3 and RNA Pol II (D) from ChIP-seq, standard CUT&RUN, and B cell-modified CUT&RUN experiments for H3K4me3 (n=3, 1, 7, respectively), IgG (n=1, 1, 7, respectfully), and RNA Pol II (n=1, 1, 7, respectively). Datasets for ChIP-seq, original, and B cell-optimized CUT&RUN are indicated by the prefixes CS-, OG-, and S-, respectively, and groups by target (H3K4me3, IgG, or RNA Pol II) are indicated by the colored bars above. G-H: PCA analysis of peaks from all normalized datasets. (G) or differential binding between H3K4me3 and RNA Pol II (H). The solid oval indicates the H3K4me3 datasets, the dashed oval indicates the RNA Pol II datasets, and the fine dotted oval indicates the IgG datasets. Differential sites are those with an FDR </= 0.05. (TIF) [file pone.0322139.s007.tif]

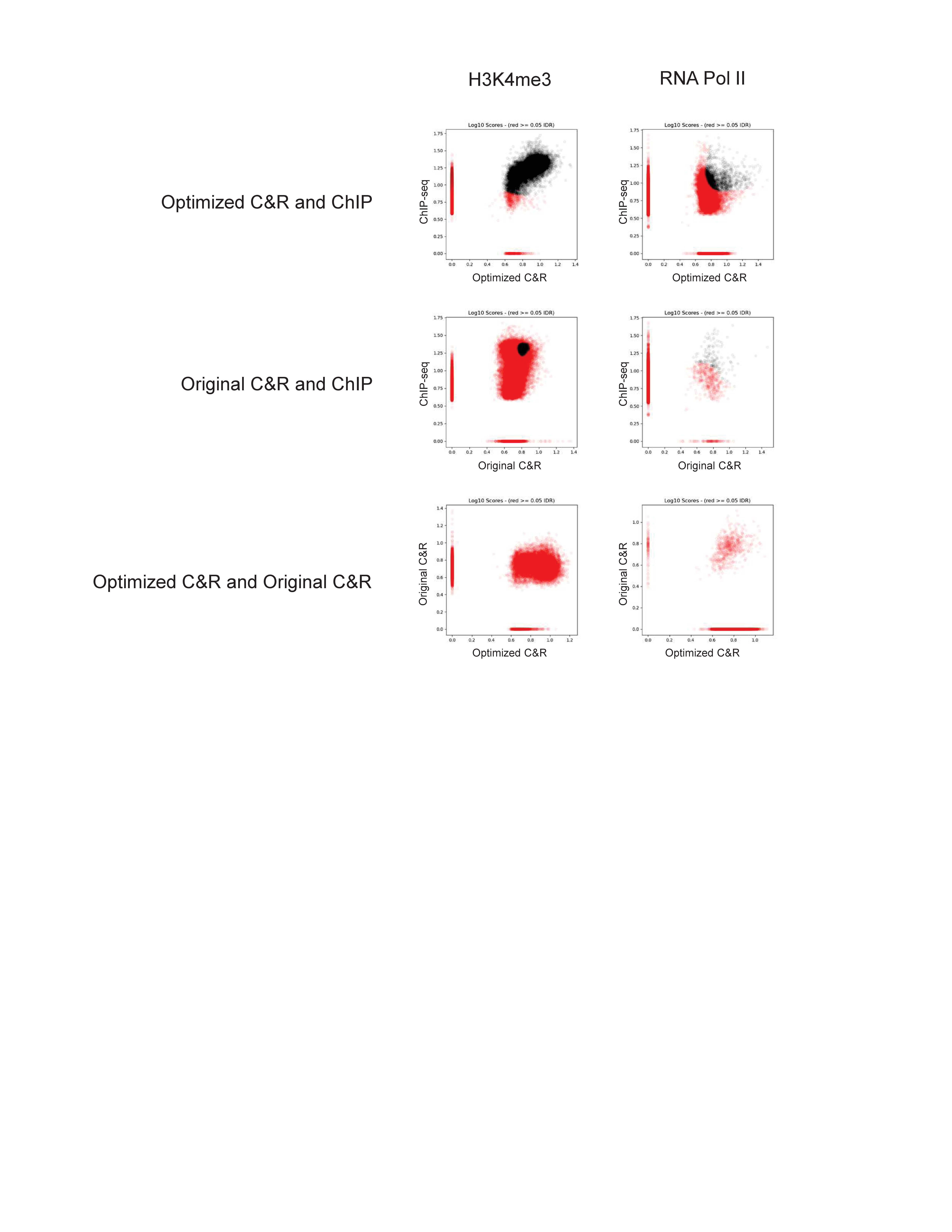

Supplement: S8 Fig — Peaks were identified from samples for H3K4me3 (left) or RNA Pol II (right) using Macs2 with IgG samples used as control and the setting “–broad”. IDR analysis was performed using the settings “--use-nonoverlapping-peaks” and “--use-best-multisummit-IDR”. Comparisons are Optimized CUT&RUN (C&R) (top), original CUT&RUN (C&R) (middle), and ChIP-seq (bottom). Black indicates an IDR < 0.05 and axes are log(10) scores. (TIF) [file pone.0322139.s008.tif]

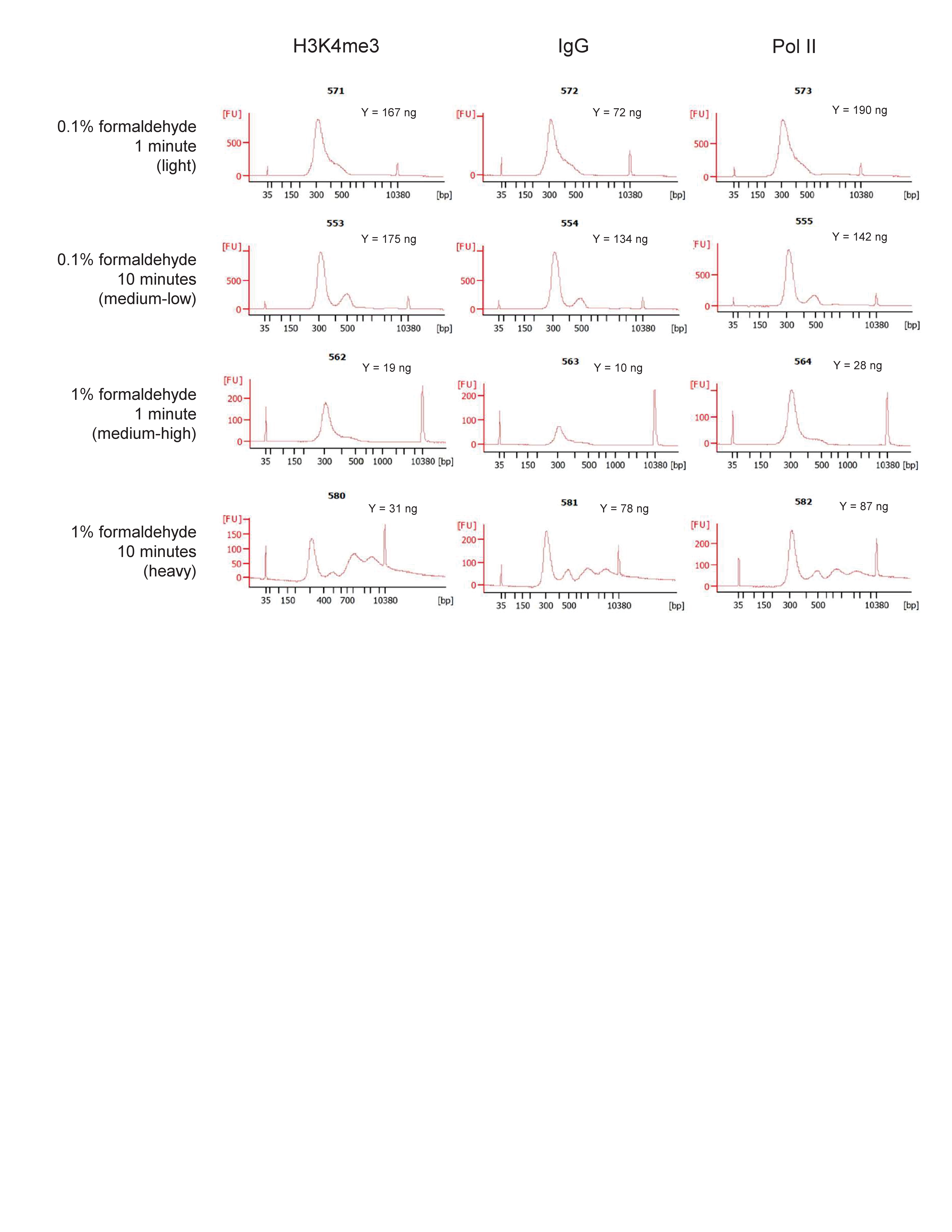

Supplement: S9 Fig — CUT&RUN was performed at 500k cells each sample under one of the four fixation conditions (light, medium-low, medium-high, heavy). Resulting DNA fragments were used to prepare libraries for sequencing. Library size and quantity was obtained using the Bioanalyzer High Sensitivity DNA kit (Agilent) and Qubit 1x dsDNA High sensitivity kit (Invitrogen) (respectively). Traces from the Bioanalyzer are shown while yield (calculated from size data from the Bioanalyzer and concentration from the Qubit) are displayed at the top right of each trace. (TIF) [file pone.0322139.s009.tif]

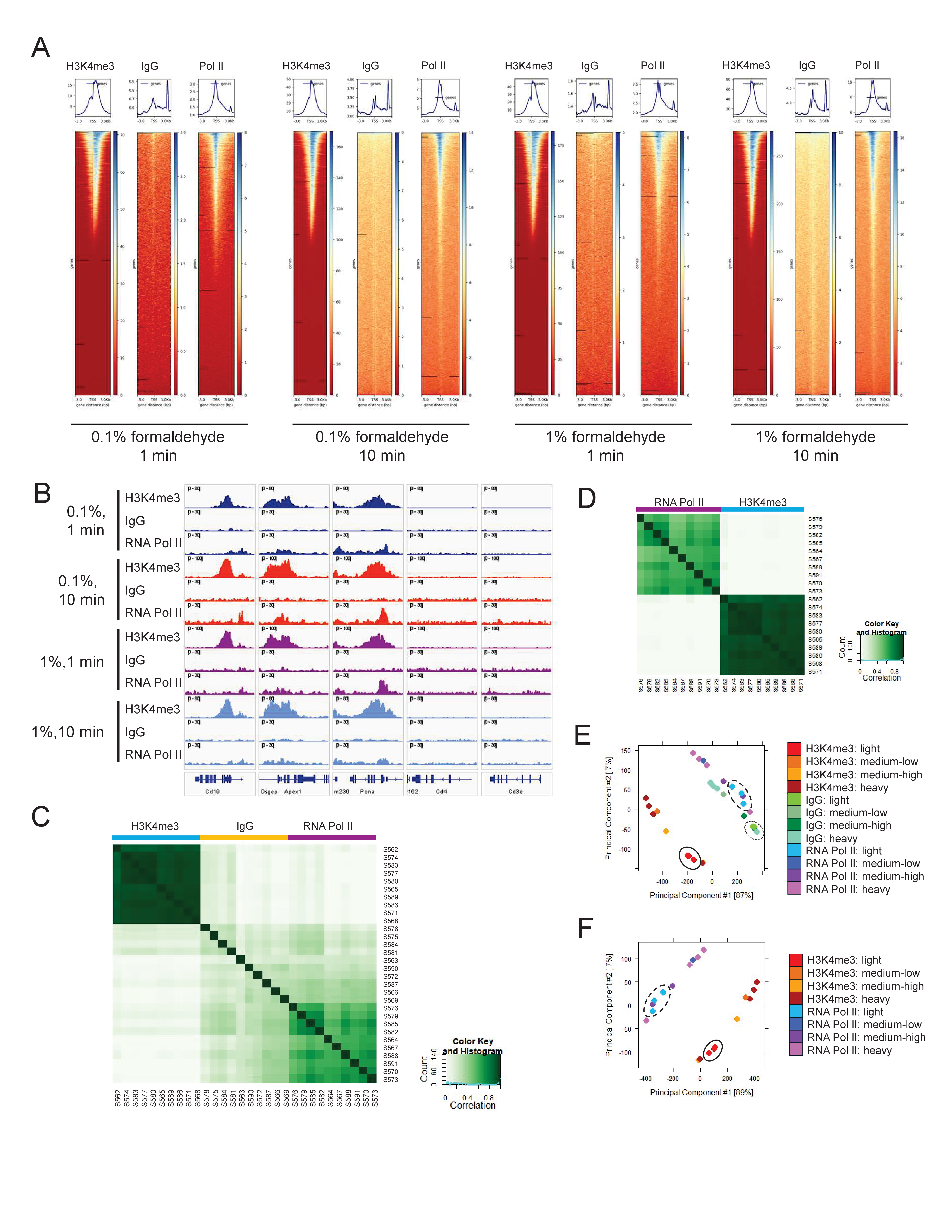

Supplement: S10 Fig — B: IVG tracks for the four fixation conditions at select B cell (Cd19), DNA repair/CSR genes (Apex1, Pcna), and non-B cell genes (Cd4, Cd3e). C-D: Affinity matrices comparing cross-correlation for all normalized (C) or differentially bound (D) sites for group 2 datasets under the four treatment conditions. E-F: PCA plots for group 2 datasets at all normalized (E) or differentially bound (F) sites. Differential sites are those with an FDR </= 0.05. (TIF) [file pone.0322139.s010.tif]

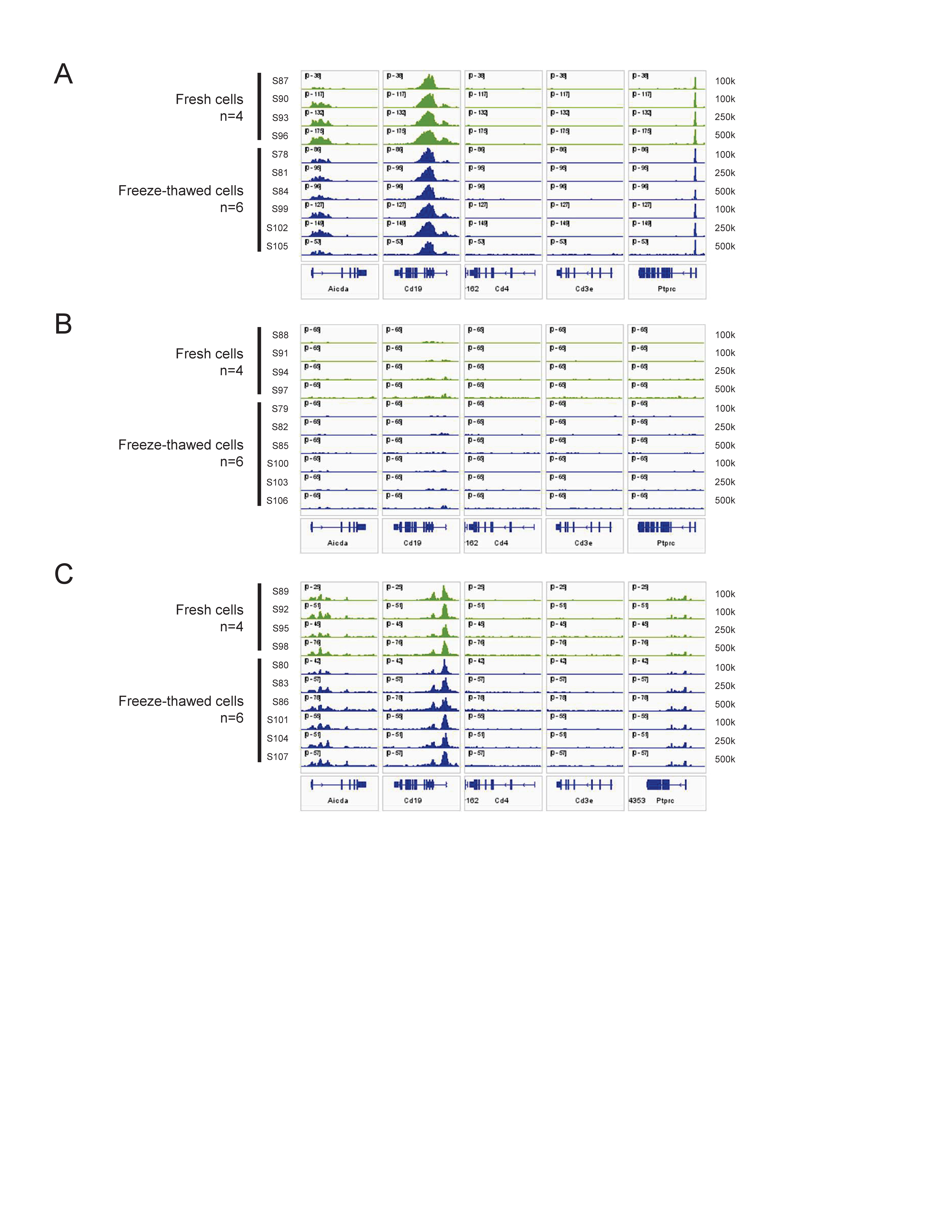

Supplement: S11 Fig — A and B: Tracks for fresh (n=4, green) and frozen (n=6, blue) samples at select genes for HK4me3 (A), IgG (B) and RNA Pol II (C). Sample IDs are listed to the left of each trace (see S4 Table) while number of nuclei used is to the right. (TIF) [file pone.0322139.s011.tif]

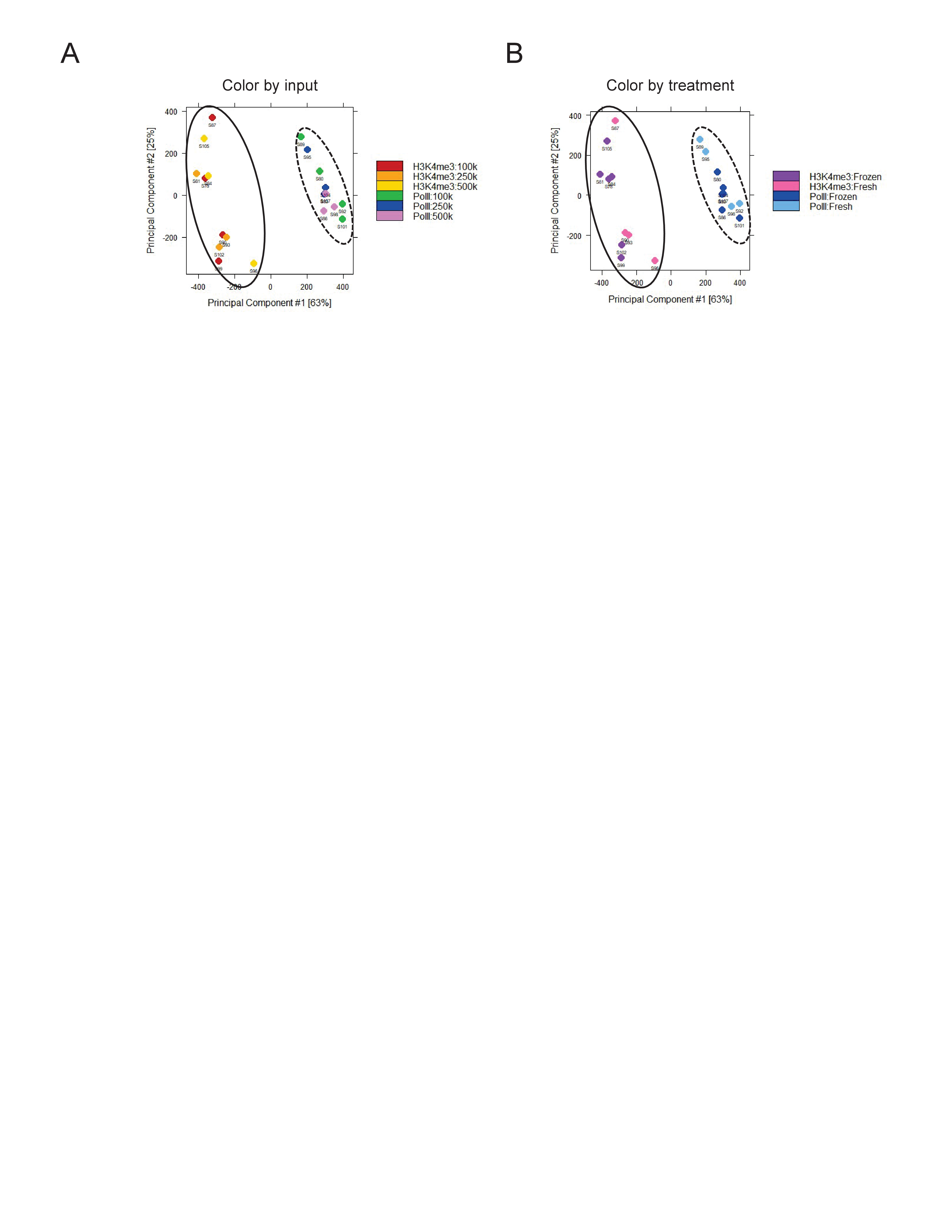

Supplement: S12 Fig — A and B: Differentially bound sites with an FDR <0.05 between the H3K4me3 and RNA Pol II sample groups were used to generate a PCA plot to visualize differences across groups. Samples are colored by input (A) or treatment (B). H3K4me3 datasets are enclosed in a solid oval while RNA Pol II datasets are enclosed in a dashed oval. (TIF) [file pone.0322139.s012.tif]
